# The Networking Brain: How Extracellular Matrix, Cellular Networks, and Vasculature Shape the In Vivo Mechanical Properties of the Brain

**DOI:** 10.1002/advs.202402338

**Published:** 2024-06-14

**Authors:** Judith Bergs, Anna S. Morr, Rafaela V. Silva, Carmen Infante‐Duarte, Ingolf Sack

**Affiliations:** ^1^ Department of Radiology Charité – Universitätsmedizin Berlin Charitéplatz 1 10117 Berlin Germany; ^2^ Experimental and Clinical Research Center a cooperation between the Max Delbrück Center for Molecular Medicine in the Helmholtz Association and Charité – Universitätsmedizin Berlin Lindenberger Weg 80 13125 Berlin Germany; ^3^ Corporate Member of Freie Universität Berlin and Humboldt‐Universität zu Berlin ECRC Experimental and Clinical Research Center Charité – Universitätsmedizin Berlin Charitéplatz 1 10117 Berlin Germany; ^4^ Max Delbrück Center for Molecular Medicine in the Helmholtz Association (MDC) Robert‐Rössle‐Straße 10 13125 Berlin Germany

**Keywords:** brain mechanical networks, cerebral vasculature, extracellular matrix, in vivo viscoelasticity, magnetic resonance elastography, neurons, sonoelastography

## Abstract

Mechanically, the brain is characterized by both solid and fluid properties. The resulting unique material behavior fosters proliferation, differentiation, and repair of cellular and vascular networks, and optimally protects them from damaging shear forces. Magnetic resonance elastography (MRE) is a noninvasive imaging technique that maps the mechanical properties of the brain in vivo. MRE studies have shown that abnormal processes such as neuronal degeneration, demyelination, inflammation, and vascular leakage lead to tissue softening. In contrast, neuronal proliferation, cellular network formation, and higher vascular pressure result in brain stiffening. In addition, brain viscosity has been reported to change with normal blood perfusion variability and brain maturation as well as disease conditions such as tumor invasion. In this article, the contributions of the neuronal, glial, extracellular, and vascular networks are discussed to the coarse‐grained parameters determined by MRE. This reductionist multi‐network model of brain mechanics helps to explain many MRE observations in terms of microanatomical changes and suggests that cerebral viscoelasticity is a suitable imaging marker for brain disease.

## Graphical abstract

A reductionist view of brain anatomy assumes that there are four basic networks that play a major role in shaping the in vivo brain mechanical properties: neurons, glial cells, extracellular matrix, and vasculature. This physics‐based model explains brain elastography findings, shedding light on the relationship between microanatomy and macroscopic biomechanical changes, which can be leveraged as clinical imaging markers.

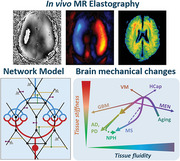

## 1 Introduction

Organ function is tightly linked to tissue microstructure. In the brain, myriads of neurons continuously receive and transmit signals for processing the information arriving from within the body or taken up by our senses. To accomplish this task, the neural network acts in concert with glial cells, blood vessels, and extracellular components. These four components form an intricate functional network that collectively determines the shape, consistency, and thus the mechanical properties of the brain.^[^ ^1^, ^2^ ^]^ Consequently, the material properties of the brain – among the softest and most viscous in the human body – emerge from all the structures that maintain neuronal function and integrity.^[^ ^3^, ^4^ ^]^

Neurons unceasingly monitor their microenvironment through their growth cones and adapt to new information or environmental influences. This unique feature of neuroplasticity, which underlies learning and memory, requires flexible cellular networks with optimized pathways for signal transduction and nutrient provision across multiple scales. Nature efficiently forms such networks by providing them with a hierarchically ordered topology,^[^ ^5^ ^]^ which, to a certain extent, is scale‐free.^[^ ^6^ ^]^ To ensure their flexibility, cellular networks must be sparsely embedded in a mechanically compliant matrix.^[^ ^7^ ^]^ Blood vessels and perivascular spaces give the brain a porous character^[^ ^8^ ^]^ for optimal provision of nutrients and drainage of metabolites. Overall, these features of hierarchy, sparsity, compliance, and flexibility contribute to the unique combination of fluid‐like viscous and solid‐elastic properties. Which of these traits predominates depends on the observer, as the time scale and amplitude of the mechanical test determine the brain's mechanical response.^[^ ^9^, ^10^ ^]^

Under normal circumstances, the brain cannot be palpated, which in part explains why only little information is available on either physiological or pathological changes of its mechanical properties in vivo. However, elastography, an emerging medical imaging technique, depicts viscoelastic parameters in soft tissues in vivo, using shear waves, and has the potential for clinical diagnosis of central nervous system (CNS) diseases.^[^ ^11^, ^12^ ^]^ Elastography permits quantification of parameters such as complex shear modulus, shear wave speed, viscosity, shear strain, and volumetric strain.^[^ ^13^ ^]^ Most in vivo elastography studies in the brain have been performed using magnetic resonance elastography (MRE).^[^ ^14^, ^15^, ^16^, ^17^, ^18^, ^19^ ^]^ In addition, ultrasound elastography (USE)^[^ ^20^, ^21^, ^22^, ^23^, ^24^, ^25^ ^]^ has been introduced for measuring brain stiffness. Unlike MRE, cerebral USE cannot (yet) resolve in detail the anatomical distribution of brain stiffness but provides instantaneous feedback on short time scales.^[^ ^26^ ^]^ Invasive methods such as atomic force microscopy (AFM) and shear rheometry have been established to investigate brain mechanical properties from the surface.^[^ ^27^ ^]^ Studies performed with these techniques have linked mechanical parameters with microstructures and provide evidence of the relevance of mechanobiology for proper brain physiology.^[^ ^10^, ^28^ ^]^ Combining these insights with noninvasive in vivo MRE and USE in a variety of neurological conditions reported in the literature may provide unprecedented insights into the interplay between microanatomy and macroscopic material properties of the brain. Consequently, this article complements the large body of literature in this area^[^ ^14^, ^16^, ^19^, ^29^ ^]^ by discussing the key structural components of the brain from the perspective of in vivo brain mechanics. We aim to understand how different elements of brain structure act in concert in forming the mechanical backbone of the brain and how this mechanical signature changes in disease. Ultimately, a mechanistic understanding of the interplay between tissue mechanics and brain processes may be transformed into novel biomarkers or therapeutic approaches for neurological disorders.

## 2 Brain Mechanical Elements

**Figure 1:**
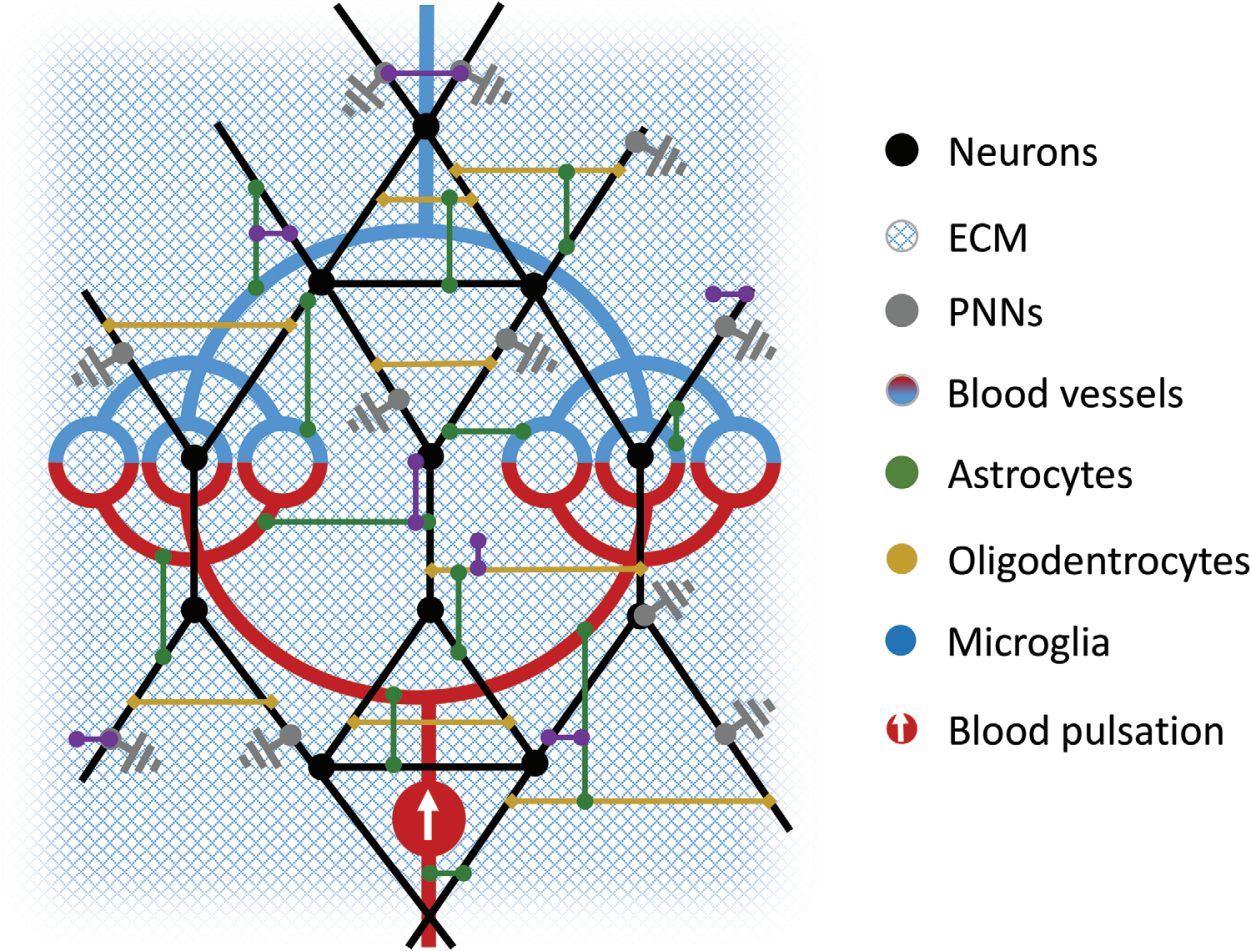
Sketch of the reductionist model of the basic multiscale, multi‐element networks that shape the composite mechanical properties of the brain. Schematic illustration of the brain's structural arrangement of vessels with fluid transport, glial cells, neurons, and the extracellular matrix (ECM), including perineuronal nets (PNNs) and various cell adhesion molecules (CAMs) such as integrins or proteoglycans. The four essential networks that we consider to be relevant for the overall mechanical response of the brain are shown. The two cellular networks are made up of neurons and glial cells. The latter establishes the mechanical links between neurons (oligodendrocytes) and between neurons and the vascular network (astrocytes). The cellular networks are anchored to the ECM via PNNs^[^ ^3^ ^]^ and CAMs.^[^ ^30^ ^]^ Softening of brain tissue can be attributed either to the loss of integrity within one of these four networks or to a loss of crosslinking between them. Conversely, the addition of new elements or reinforcement of individual elements including crosslinks or an increase in vascular pressure will result in an overall strengthening of brain networks and cause brain stiffening. Changes in the tissue's solid‐fluid behavior are mainly related to ECM‐water interactions, fluid content, cell motility, and friction.^[^ ^31^, ^32^ ^]^

In a reductionist view of brain anatomy, we have identified four basic networks that play a major role in shaping the viscoelastic properties of the brain: neurons, glial cells, the extracellular matrix (ECM), and the vasculature (**Figure** **1**). Although this categorization oversimplifies brain anatomy from the perspective of biological processes, the mechanical model of four dynamically interconnected networks, all ordered in a hierarchy of geometries, and responding to deformation in a nonlinear fashion over a wide range of lengths and time scales, is overwhelmingly complex.

**Figure 2:**
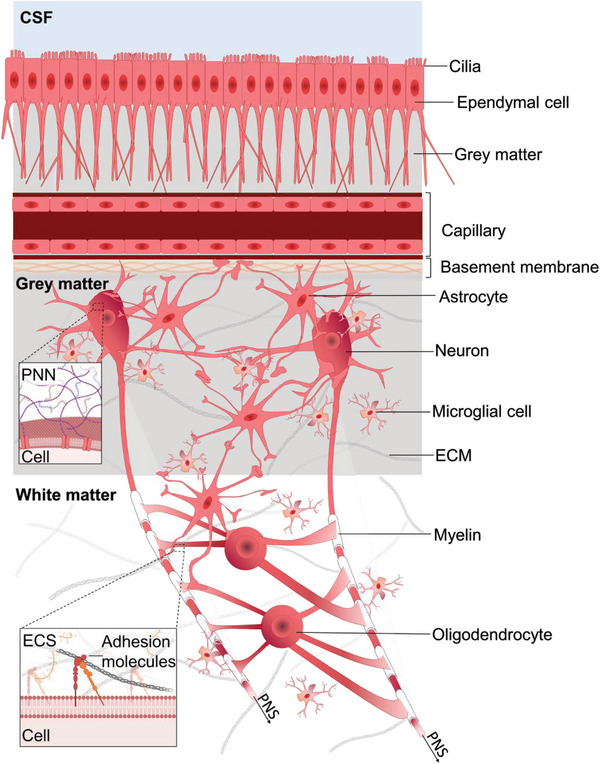
Components of CNS tissue. Schematic representation of the intricate cellular architecture that maintains the structure and function of the brain. Cerebrospinal fluid (CSF), a colorless fluid, circulates from the lateral ventricles to the third and fourth ventricles, providing essential nutrients to support brain homeostasis and growth factors during brain development.^[^ ^48^ ^]^ Motile cilia, which line the apical surfaces of ependymal cells, stimulate CSF circulation by cilia beating. The blood‐brain barrier, formed by interconnected cerebral capillary endothelial cells with tight junctions and the glia limitans, acts as a protective barrier,^[^ ^49^, ^50^ ^]^ preventing the free transport of potentially harmful substances into the brain while facilitating the provision of nutrients and removal of metabolites.^[^ ^51^ ^]^ Neurons, the primary cell type in the brain, form a network through synaptic connections.^[^ ^52^ ^]^ Other nonneuronal brain cells include glial cells, endothelial cells lining the blood vessels, and ependymal cells lining the ventricles. Glial cells, the second most common cell type in the brain, can be further categorized into oligodendrocytes (responsible for myelin formation around neuronal axons),^[^ ^44^ ^]^ microglia (immune cells of the brain),^[^ ^53^ ^]^ and astrocytes (forming borders along all blood vessels and meningeal surfaces).^[^ ^53^ ^]^ Myelin, which wraps around some axons in the CNS, serves as an electrical insulator for efficient signal propagation and provides structural support and protection.^[^ ^38^, ^39^ ^]^ The high lipid content of myelin gives white matter its characteristic bright appearance, as it consists mainly of myelinated axons. Gray matter, on the other hand, contains neuronal somas, unmyelinated axons, and dendrites.^[^ ^53^ ^]^ The CNS transmits signals to the peripheral nervous system (PNS) through descending nerve tracts. Cells are anchored to the extracellular space (ECS) via perineuronal nets (PNN) and adhesion molecules).

The two cellular networks are made up of neurons and glial cells (astrocytes, oligodendrocytes, and microglia). From a mechanical perspective, the glial network crosslinks neurons and establishes mechanical bridges with all other brain components including neurons and the vascular network (**Figure** **2**). For example, beyond the classical view on connections between neurons and astrocyte and neurons and oligodendrocytes, it has been shown that astrocyte‐oligodendrocyte interactions regulate CNS regeneration.^[^ ^33^ ^]^

**Figure 3:**
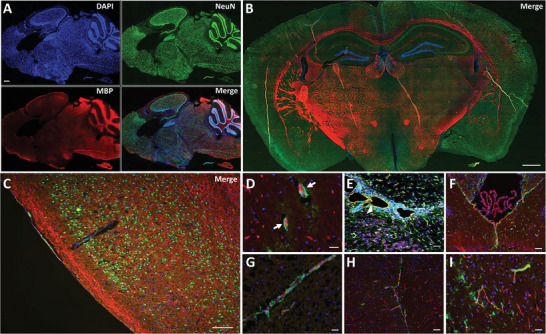
Neuron and myelin distribution and interaction between astrocytes and endothelial cells in the mouse brain. A) Immunofluorescence staining of the murine brain in a sagittal view, showing cell nuclei in blue (DAPI), neurons in green (NeuN), and myelin in red (MBP). Neuron density varies among the different brain regions. In the human brain, most neurons are located within the cerebellum (in the granular cell layer), while the cerebral cortex contains relatively fewer neurons,^[^ ^34^ ^]^ as is also seen here in the mouse brain. Scale bar: 500 µm, magnification 10×. B) Immunofluorescence staining of the murine brain in a coronal view, showing cell nuclei in blue (DAPI), neurons in green (NeuN), and myelin in red (MBP). Neuronal density varies across different brain regions (cerebral cortex, hippocampus, thalamus, hypothalamus, and striatum). Scale bar: 500 µm, magnification 10×. C) Immunofluorescence staining of the cerebral cortex showing neurons in green (NeuN), myelin in red (MBP), and cell nuclei in blue (DAPI). Neuron density varies across different layers^[^ ^54^ ^]^ and regions^[^ ^55^ ^]^ within the cerebral cortex, with the highest concentration observed in the primary visual cortical area, V1, particularly at the site of central visual representation.^[^ ^54^ ^]^ Neurons (NeuN – green), myelin (MBP – red), cell nuclei (DAPI – blue). Scale bar: 100 µm, magnification ×20. D) Immunofluorescence staining of the cerebral cortex displaying astrocytes in green (GFAP), endothelial cells in red (CD31), and cell nuclei in blue (DAPI). Image shows astrocytes wrapped around small arterioles and capillaries,^[^ ^53^ ^]^ which are formed by endothelial cells (white arrows). Scale bar: 20 µm, magnification 40×. E) Imaging mass cytometry image of brain tissue from an EAE mouse showing astrocytes in green (GFAP), endothelial cells in yellow (CD31), neurons in pink (NeuN), and cell nuclei in blue (Histone‐H3). There is detachment of glial network elements, shown here as degraded astrocyte‐vessel connections, due to neuroinflammation (white arrowhead).^[^ ^56^ ^]^ Scale bar: 50 mm. Image adapted with permission from.^[^ ^57^ ^]^ F–I) Immunofluorescence staining displaying astrocytes in green (GFAP), endothelial cells in red (CD31), and cell nuclei in blue (DAPI). Astrocytes are densely present at the interfaces between brain parenchyma and nonneuronal cells, forming boundaries along all blood vessels and meningeal surfaces.^[^ ^42^ ^]^ c) Scale bar: 50 µm, magnification 20×, d‐e) Scale bar: 20 µm, magnification 40×. a & c‐f) Astrocytes (GFAP – green), endothelial cells (CD31 – red), cell nuclei (DAPI – blue). b) Astrocytes (GFAP – green), endothelial cells (CD31 – yellow), neurons (NeuN – purple), cell nuclei (Histone H3 – blue).

In general, an adult human brain is estimated to contain about 171 billion cells: approximately half of those are neurons.^[^ ^34^ ^]^ The rodent brain approximately contains 109 million cells, of which two thirds, or 70 million, are neurons.^[^ ^35^ ^]^ Neuron density varies widely across the different brain regions (Table S1, Supporting Information; **Figure** **3**), with most neurons located within the cerebellum, while the cerebral cortex contains relatively fewer neurons (Table S1, Supporting Information; Figure 3).^[^ ^34^, ^36^ ^]^ The largest part of the cortex consists of gray matter (GM), which contains mostly somas, some unmyelinated axons as well as dendrites of neurons.^[^ ^37^ ^]^ Some of the axons in the CNS are enwrapped by myelin in a discontinuous layer‐like fashion, making up the white matter (WM).^[^ ^38^ ^]^ It has been discussed that myelin, besides being an electrical insulator, acts as a microstructural scaffold^[^ ^39^ ^]^ and protector against physical forces.^[^ ^38^ ^]^ WM is found mostly at the surface of the spinal cord and in deeper brain regions^[^ ^40^ ^]^ and constitutes about half of the total brain volume.^[^ ^41^, ^42^ ^]^ The majority of nonneuronal cells in the brain are glial cells.^[^ ^35^ ^]^ Gilal cells can be subdivided into microglia and macroglia. Microglia account for ≈10% of the CNS cell population.^[^ ^43^ ^]^ Astrocytes are the most abundant type of macroglia^[^ ^44^ ^]^ and are present throughout the brain, particularly at the interfaces between CNS parenchyma and nonneuronal cells, where the perivascular astrocyte endfeet ensheath blood vessels and meningeal surfaces^[^ ^45^ ^]^ (Figure 3D–I) and provide water, ions, and energy to the neurons.^[^ ^46^ ^]^ Another important type of macroglia is oligodendrocytes, the myelinating cells of the CNS.^[^ ^47^ ^]^

Cellular networks are embedded in the ECM, which itself is a macromolecular network immersed in extracellular fluid (ECF). The brain's ECF consists of interstitial fluid (ISF) and CSF and accounts for 15 to 30% of the extracellular volume fraction (ECV) of the normal adult brain.^[^ ^58^ ^]^ This is markedly higher than in other organs such as healthy human liver, which has an ECV of only 0.5%.^[^ ^59^ ^]^ ISF and CSF have important roles for cell function by providing nutrients and removing waste products in addition to their mechanical function of cushion and support.^[^ ^60^ ^]^ Furthermore, CSF in ventricles and subarachnoid spaces provides buoyancy and shock absorbance to the entire brain.^[^ ^61^ ^]^ CSF circulation is stimulated by the beating of motile cilia lining the apical surfaces of ependymal cells. The colorless CSF is separated from the blood pool by the blood‐CSF barrier (BCB). The highly compliant and pronounced viscous‐fluid material properties of brain ECM, supported by ISF and CSF infiltration, provide neurons with the flexibility to remodel synapses and connections.^[^ ^58^ ^]^ These distinctly soft and viscous‐fluid properties of brain ECM compared with other organs are likely attributable to its unique composition of glycosaminoglycans (GAGs), glycoproteins (GPs), and proteoglycans (PGs), while fibrillar collagens and fibronectin are sparse (except in the meninges and blood vessels).^[^ ^62^ ^]^ Neurons are anchored to the ECM by cell adhesion molecules (CAMs) such as integrins.^[^ ^30^ ^]^ Another brain‐specific crosslink between cells and ECM is provided by perineuronal nets (PNNs) – lattice‐like structures made up of PGs and GAGs that intertwine neuronal cell bodies, dendrites, and parts of axons of specific subsets of neurons.^[^ ^62^, ^63^ ^]^ PNNs^[^ ^3^ ^]^ and cell adhesion molecules^[^ ^30^ ^]^ transmit mechanosensory signals into the cytoskeleton of neurons and, thus, convert mechanical forces into biochemical or electrical signals.^[^ ^30^ ^]^ PNNs also form channel‐like structures for extracellular fluid transport and drainage, which makes them important for the microporous properties of brain tissue.^[^ ^64^, ^65^, ^66^ ^]^

Another major contributor to the poroelastic nature of brain tissue is blood. Brain mechanical properties are affected by fluid pressure in the blood vessels, the blood perfusion rate, and the geometry of the intricate vascular neuroanatomy contributes to brain mechanical properties. While vascular networks are often depicted as tree‐like structures, cerebral vessels have a very specific topology that leads to obvious redundancies.^[^ ^67^ ^]^ As early as 1872, Heubner recognized that the cerebrovascular system is full of anastomoses (shortcuts) both within the arterial and venous systems and between arteries and veins at all levels.^[^ ^68^ ^]^ Henceforth, we refer to the vasculature as the fourth network in our model. Human brain tissue has a high metabolic demand, accounting for ≈20% of total body oxygen consumption.^[^ ^69^ ^]^ The major site of blood‐CNS exchange is the blood‐brain barrier (BBB), which is primarily made up of tightly interconnected cerebral capillary endothelial cells.^[^ ^49^, ^50^ ^]^ Microvessels, including capillaries, arterioles, and venules, maintain the BBB with continuous tight junctions, whereas blood vessels near the ventricular system and in the choroid plexus do not.^[^ ^70^ ^]^ Detailed information on the volumes, diameters, amounts of endothelial cells, and densities of the brain's vascular components is compiled in Table S1 (Supporting Information). Cerebral blood flow (CBF) is tightly autoregulated to ensure a constant blood flow under normal physiological variations such as changes in heart rate and blood pressure, blood oxygenation, CO_2_, and nutrient supply.^[^ ^71^ ^]^ Alterations in CBF have been observed in many neurological disorders including schizophrenia and Alzheimer's disease.^[^ ^71^ ^]^ As the density of microvessels in brain tissue correlates with metabolic activity, vascular supply varies between the different regions of the brain, most markedly between GM and WM.^[^ ^72^ ^]^ Microvessel density is higher in GM than in WM due to the higher synaptic activity and metabolic demand of neurons.^[^ ^51^ ^]^ Arterioles, capillaries, and venules in the brain are enveloped by the endfeet processes of astrocytes.^[^ ^73^, ^74^ ^]^ These endfeet form the outer wall of a perivascular space that surrounds the vasculature in different brain regions.^[^ ^74^, ^75^, ^76^ ^]^ Immunostainings presented in Figure 3 show the spatial proximity of blood vessels and astrocytes and therewith the interconnectedness of the different networks that form the brain's mechanical scaffold. The interconnectivity of this multi‐element neural network, as illustrated in Figure 1, explains why brain tissue softens when the crosslinks between different network elements are weakened and why their reinforcement or the formation of new links leads to overall stiffening.

## 3 A Brief Summary of Elastography Techniques Suitable for In Vivo Studies of Brain Mechanics

**Figure 4:**
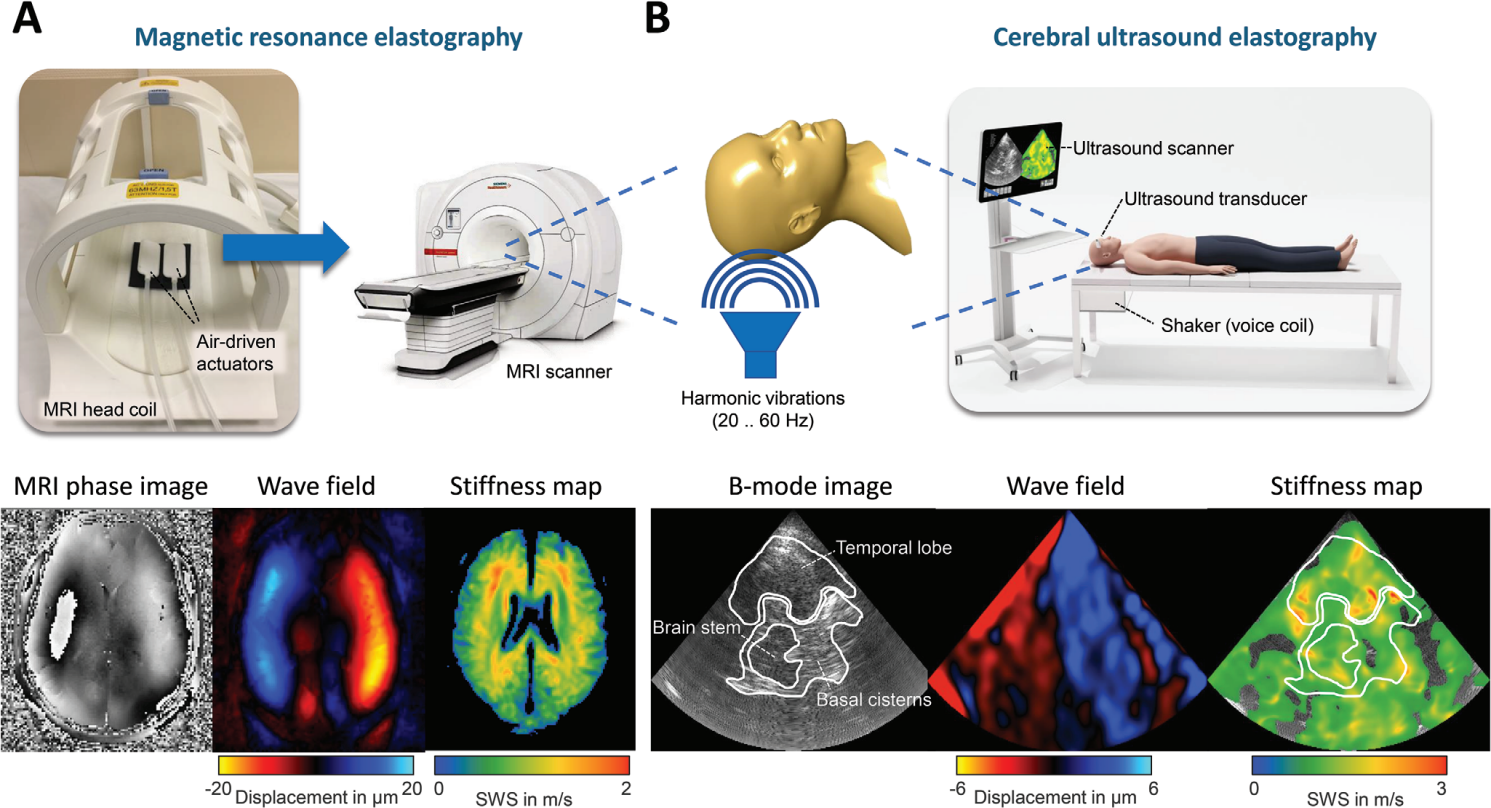
Technical setup, image acquisition, and postprocessing of brain elastography based on magnetic resonance imaging and medical ultrasound. A) Typical setup of cerebral magnetic resonance elastography (MRE). Mechanical vibration frequencies are set at the MRI console. A pressure control unit is used to transmit vibrations into the head through two actuators integrated into the radiofrequency (RF) head coil. Shear wave speed maps (SWS, in ms^−1^), a surrogate for tissue stiffness, are reconstructed using wavenumber‐based (*k*‐)MDEV inversion^[^ ^79^ ^]^ with brain‐adapted preprocessing.^[^ ^80^ ^]^ In short, phase images are acquired by multifrequency MRE and wave images are processed using wave number reconstruction at the different frequencies, which is followed by amplitude‐weighted averaging. Next, Fourier decomposition over time is performed, and directional spatial filters are applied. B) Technical setup and image acquisition using cerebral ultrasound elastography (USE). The ultrasound probe is positioned over the temporal bone window for transcranial ultrasound. Multifrequency vibrations are transmitted into the head via a vibration plate integrated into the patient bed.^[^ ^21^ ^]^ Wave image acquisition and postprocessing, as described in (A), are integrated into the elastography computer, which automatically generates stiffness maps in real‐time.

Elastography uses medical imaging techniques to encode mechanical strain.^[^ ^13^ ^]^ If the stress causing the deformation is well known, the elastic modulus can be inferred from strain images. Static mechanical stresses are difficult to measure noninvasively in biological tissue. Oscillatory stresses, on the other hand, propagate through bulk tissue at a speed that depends on tissue stiffness. Most elastography techniques therefore capture tissue vibrations induced by external vibration or acoustic pressure pulses to determine mechanical parameters.^[^ ^77^ ^]^ Cerebral elastography requires robust wave excitation to penetrate the skull and induce strain fields in the brain. Typically, this is achieved by time‐harmonic vibrations of the head with frequencies in the range of 10 to 80 Hz in humans and up to 2 kHz in mice. The resulting strain fields are encoded by phase‐contrast magnetic resonance imaging (MRI)^[^ ^78^ ^]^ or medical ultrasound (US)^[^ ^24^ ^]^ (**Figure** **4**). Wave inversion algorithms are then applied to convert the shear wave fields into property maps. Sets of mechanical parameters measured by elastography include storage modulus, shear wave velocity, and Young's modulus as stiffness‐related parameters.^[^ ^13^ ^]^ Viscosity can be parameterized by elastography based on loss modulus, wave attenuation, penetration rate, dispersion, loss angle, or fluidity.^[^ ^13^ ^]^ Although slower than USE, MRE provides more detailed maps of stiffness and viscosity‐related parameters of brain tissue. Today, cerebral MRE is a versatile technique on the verge of clinical translation. Brain USE is still more experimental and requires further validation in clinical trials.

## 4 Mechanical Properties of the Brain In Vivo

What would the brain feel like in an intact skull if we could palpate it? Any type of mechanical test exerts a stress on the investigated material that induces deformation and thus allows measurement of the material's intrinsic resistance to that strain.^[^ ^81^ ^]^ Since the bones that form the skull prevent manual palpation of the brain, we can only resort to the following *gedankenexperiment* of palpating a brain confined in its normal environment.

Palpation induces shear strain that probes shear modulus. Note that palpation, even with axial displacement, gently shifts layers of tissue against each other, i.e., causing shearing rather than compression, which would be felt as pain. If the material is a monophasic solid and in the regime of small deformations, shear modulus is independent of compression modulus since shear strain is volume‐conservative. The situation is different for a sponge‐like material, where liquid is displaced by shear deformation, resulting in local volumetric strain.^[^ ^82^, ^83^ ^]^ The intrinsic resistance to this type of deformation is the effective shear modulus of a poroelastic medium.^[^ ^84^ ^]^ In such a scenario, the shear modulus of the solid phase is related to hydraulic conductivity, that is, the ease with which the liquid phase can be squeezed through channels and pores.^[^ ^85^, ^86^ ^]^ The poroelastic model applies here because brain tissue consists of several fluid phases that permeate the tissue matrix. In addition to blood and ISF, the brain comprises CSF, which lubricates abundant sulci and ventricles, making the brain slippery and soft at larger scales.^[^ ^87^ ^]^ Thus, the slip boundaries contribute to the extremely soft properties of the brain as sensed by our fingers.^[^ ^10^, ^88^ ^]^ Conversely, the effective shear modulus increases with perfusion pressure in cerebral arteries and arterioles.^[^ ^89^ ^]^ Moreover, pressurized vessel walls can contribute to an overall increase in brain shear modulus due to hyperelastic (nonlinear) expansion.^[^ ^90^ ^]^ Similarly, brain tumors that grow in a displacing manner or accumulation of encapsulated fluids in edema exert solid stress on the surrounding tissue, stretching tissue fibers and, due to nonlinear effects such as strain stiffening, increasing the effective shear modulus.^[^ ^91^ ^]^ In general, compressive stress in the brain causes an increase in intracranial pressure (ICP) since brain volume cannot expand much within the cranial cavity, as stated by the Monro‐Kellie doctrine.^[^ ^92^ ^]^ This pressure increase translates into an increase in the effective shear modulus by altered boundary conditions at numerous fluid‐to‐solid interfaces in the brain, which are related to changes in pore size and pore pressure including hyperelastic responses of vessel walls.^[^ ^93^, ^94^ ^]^

**Figure 5:**
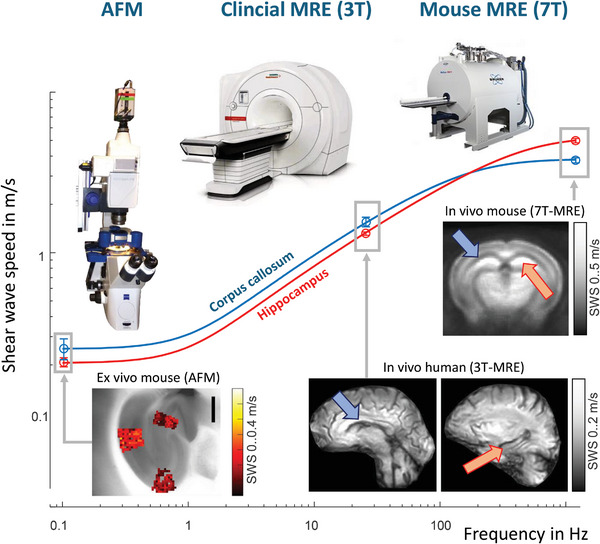
Mechanical response of brain tissue to quasi‐static, low‐frequency stimulation, and high‐frequency stimulation. Differences in the mechanical response of the hippocampus (orange arrow) and the corpus callosum (blue arrow) to stimulation at different frequencies ranging from quasi‐static methods such as atomic force microscopy (AFM) to low‐frequency stimulation using clinical 3‐Tesla magnetic resonance elastography (MRE), to high‐frequency stimulation as performed with preclinical 7‐Tesla MRE. Shear wave speed as a proxy of stiffness was measured by MRE while AFM measured Young's modulus which was converted to SWS based on the assumption of incompressible tissue properties. The disparity between low‐frequency data (in the range of 30 Hz) showing corpus callosum to be stiffer than hippocampus and high‐frequency data (in the range of 1000 Hz) showing the opposite can be reconciled by a standard three‐parameter material model that combines two elastic elements with one viscous element.^[^ ^110^ ^]^ Figure adapted with permission from.^[^ ^110^ ^]^

Pressure‐related effects that lead to a rise in apparent brain stiffness disappear when the brain is removed from the skull and disconnected from arterial perfusion.^[^ ^95^ ^]^ Fresh, *ex vivo* brain is so soft that it deforms by gravity alone and cannot hold its own shape, even if the surrounding connective tissue of the pia mater is intact.^[^ ^88^ ^]^ This ultrasoft behavior is related in part to the presence of fluid compartments at the macroscopic level, such as CSF in sulci, fluid blood in vessels, and ISF in tissue channels and pores. The dissection of tissue and disintegration of vessels allow fluids to move freely on the time scales of testing.^[^ ^96^ ^]^ Mechanical test methods typically select bulky brain tissue excluding fluid areas in sulci and larger vessels.^[^ ^97^, ^98^ ^]^ This is one reason why investigators have reported solid tissue values in the range of hundreds of Pascals up to several Kilopascals,^[^ ^27^, ^96^, ^99^ ^]^ which is higher than what we would expect from palpation.^[^ ^88^ ^]^ Another reason for disparate stiffness values is the dynamic range of the test method, as mentioned above, which is between 10 Hz in humans and 2 kHz in mice.^[^ ^100^ ^]^ The faster the tissue is deformed (i.e., the higher the frequency with which shear resistance of the tissue is measured), the higher the apparent stiffness (**Figure** **5**). The reason for this dispersion of stiffness is viscosity.^[^ ^101^, ^102^, ^103^ ^]^ Besides stiffness dispersion, viscosity also causes damping of rapid deformation and attenuation of strain waves, which, undamped, would damage the tissue.^[^ ^81^ ^]^ Viscosity conveys useful information on the intrinsic structure of a material.^[^ ^104^ ^]^ This is illustrated in our *gedankenexperiment* by comparing the palpation of brain tissue with that of jelly. Both materials can have the same soft elasticity properties, but they feel very different, mainly because they have different viscosity properties. While brain tissue has pronounced viscous properties, jelly is almost perfectly elastic.^[^ ^105^ ^]^ On a frequency axis, jelly shows a constant stiffness, corresponding to solid behavior, whereas brain stiffness increases with a slope, the angle of which is known as the loss angle 𝜑 (of the complex shear modulus) or the powerlaw exponent α of the spring‐pot model.^[^ ^106^ ^]^ The maximum loss angle of 𝜑 = π/2 (α = 1) indicates fluid behavior, meaning that the tissue cannot store elastic energy. Thus, stiffness dispersion in the range of 𝜑 = 0 to π/2 (α = 0 to 1) indicates whether a material tends toward solid‐elastic or fluid‐viscous behavior and is therefore referred to as tissue fluidity in the literature.^[^ ^107^, ^108^ ^]^ Since tissue fluidity is determined by the ratio of loss to storage properties, a more fluid‐like tissue can have a high storage modulus as long as viscous loss, i.e., the attenuation of shear waves, dominates. An example of such counterintuitive behavior is tumors: glioblastomas have been classified as soft‐solid,^[^ ^107^ ^]^ while cancers in collagen‐rich organs typically exhibit stiff‐fluid properties.^[^ ^109^ ^]^

Collectively, the wealth of information that would be accessible by simple manual palpation of brain tissue motivates the development and application of in vivo cerebral elastography. Most elastography studies in the brain have been performed with MRE based on external harmonic frequencies in a range of 10 to 80 Hz in humans and 200 to 2000 Hz in small animals.^[^ ^14^, ^16^, ^19^, ^29^ ^]^ Because of differences in the mechanical response of biological tissues between low‐frequency stimulation and high‐frequency stimulation, values measured by MRE are difficult to compare across species and length scales (Figure 5).^[^ ^110^ ^]^ As a result, and as shown in many excellent reviews of brain MRE, published values vary widely and may not always be comparable.^[^ ^14^, ^15^, ^16^, ^17^, ^18^, ^19^ ^]^ Henceforth, we will focus on relative differences and relative parameter changes by using colloquial terms such as “stiff”, “soft”, “elastic‐solid”, and “viscous‐fluid”, with the latter two categories referring to tissue fluidity. It should be noted, however, that even relative stiffness ratios can be reversed when measured in different dynamic ranges, as shown in Figure 5.

## 5 In Vivo Viscoelasticity Changes in Health and Disease

The four interrelated networks of brain mechanics, *neurons*, *glial cells*, *vasculature*, and *ECM*  illustrated in Figure 1 contribute differently to tissue mechanics under normal and abnormal conditions. In the following sections, we review in vivo cerebral MRE and USE data reported in the literature in light of our multi‐network model.

### 5.1 Neural Network

Brain viscoelastic properties are known to be affected by both physiological processes such as aging and the presence of CNS pathology. Brain maturation correlates with an increase in stiffness, for which various factors have been proposed including changes in myelination, astrocyte distribution, and ECM, though the exact underlying mechanism remains to be elucidated. One study found accumulation of microtubular structures, myelination, cytoskeleton linkage, and cell‐matrix attachment in the maturing mouse brain to be correlated with higher stiffness values while protein expression profiles, associated with axonal organization, cell adhesion. and loss of synaptic plasticity, correlated with lower tissue fluidity (decrease in viscosity).^[^ ^111^ ^]^ The notion that biological soft tissues behave like elastic‐solid materials implies *lower* tissue fluidity when viscosity or mechanical friction within the tissue decreases, which is contrary to the behavior of fluids, where *higher* liquid fluidity is associated with lower viscosity and reduced friction among fluid particles. This again emphasizes that MRE is a solid tissue technique that measures tissue fluidity as a shear‐wave‐based property that does not hold for fluid mechanics because shear waves are not supported in liquids. Therefore, the observed reduction in tissue fluidity of the maturing brain appears to be related to a general property shift of the juvenile brain toward a more solid‐like behavior associated with the establishment of larger networks due to cellular adhesion along with a simultaneous loss of synaptic plasticity.

With regard to stiffness, these MRE findings were confirmed by the observation of stiffer properties in the adult than in the juvenile brain in a micro‐indentation study of the murine hippocampus and cerebellum.^[^ ^112^ ^]^ In the same line, an increase in stiffness, measured by transient shear wave elastography and AFM, was found in the granule cell layer and in the hilus during hippocampal maturation in mice.^[^ ^113^ ^]^ Findings in children and adolescents indicate that higher brain stiffness and lower tissue fluidity (based on the damping ratio) in certain brain areas are associated with stronger performance on specific functional tasks.^[^ ^114^, ^115^, ^116^ ^]^ For example, a stiff‐elastic hippocampal behavior was found to be associated with greater aerobic fitness and was a predictor of better relational memory recall.^[^ ^116^ ^]^ This difference might be attributed to increased neurogenesis in the hippocampus as a result of higher aerobic fitness, as has been observed in mice.^[^ ^117^ ^]^ However, neurogenesis and synaptic plasticity are controlled by ECM properties, while perfusion effects associated with brain function also influence cerebral mechanical properties. Therefore, the effects of fitness and memory function on brain viscoelastic parameters appear to result from overlapping and emergent effects, as discussed further in a separate chapter below.

Myelin provides mechanical support in brain tissue as reported by different studies across species and regions.^[^ ^38^, ^39^, ^111^ ^]^ However, at higher frequencies in the mouse brain, the myelin‐rich corpus callosum was shown to be softer than the cell‐body‐rich and highly vascularized hippocampus both in vivo and ex vivo.^[^ ^110^, ^118^ ^]^ The observation that the corpus callosum is stiffer than the hippocampus at low frequency (in the range of 30 Hz) while the opposite occurs at high frequency (in the range of 1000 Hz) can be modeled using a standard three‐parameter material model that combines two elastic elements with one viscous element (Figure 5).^[^ ^110^ ^]^ By surrounding axons, myelin reinforces network fibers, which likely explains why, in the dynamic range of human MRE, WM stiffness increases and fluidity decreases in correlation with myelin content.^[^ ^11^, ^39^, ^119^ ^]^ In addition, myelination in healthy brain tissue contributes to axonal integrity and axonal crosslinking by oligodendrocytes which apparently further contributes to overall brain stiffness. Thick bundles of myelinated axonal fibers, as in the corticospinal tract (CST), may reinforce brain tissue by their relatively high bending modulus and resistance to stretch. Such neural bundles act as waveguides to shear waves, giving rise to anisotropy of shear modulus, as suggested by findings obtained using dedicated MRE or USE techniques tailored to anisotropic wave analysis in different species including humans.^[^ ^120^, ^121^, ^122^, ^123^ ^]^ Taken together, it remains open if myelin by itself reinforces neurons or if healthy axons, which have more myelin, are stiffer due to their network integration. For example, the presence of neural progenitor cells and newly emerging neurons that are not yet fully integrated into brain networks has been observed to correlate with softer properties in the murine brain.^[^ ^124^, ^125^ ^]^ However, overabundance of new neurons in the hippocampal area following neurodegeneration induced by 1‐methyl‐4‐phenyl‐1,2,3,6‐tetrahydropyridin hydrochloride (MPTP) treatment, a neurotoxic drug used in a model of Parkinson's disease in mice, causes a transient rise in brain stiffness likely due to the temporary increase in the number of new neurons and their stiffer properties compared with background ECM.^[^ ^125^, ^126^ ^]^ Moreover, in a mouse model of stroke, neuronal density correlated positively with stiffness values.^[^ ^127^ ^]^ Higher neuron density as a contributor to elevated brain stiffness can help to explain various experimental findings including higher stiffness of younger brains versus old brains,^[^ ^29^, ^128^, ^129^, ^130^, ^131^ ^]^ female brains versus male brains,^[^ ^128^, ^131^ ^]^ better or worse relational memory recall^[^ ^116^ ^]^ and healthy brains versus brains with neurodegenerative diseases.^[^ ^132^, ^133^, ^134^, ^135^ ^]^ Indeed, all of these effects have also been observed in human brains, although the large number of possible confounding factors prevents causal conclusions to be drawn. In better controlled mouse models, brain softening correlated with a reduction in the number of neurons in Alzheimer's disease^[^ ^136^ ^]^ and with demyelination in a cuprizone mouse model.^[^ ^137^ ^]^ In patients with amyotrophic lateral sclerosis (ALS), a disease that specifically affects the motor neurons within the CST, brain softening was observed based on anisotropic parameters measured by waveguide MRE.^[^ ^120^ ^]^ Ultimately, brain mechanics changes postmortem,^[^ ^138^ ^]^ which is potentially, at least in part, related to changes in the mechanics of the neural network. Continuous MRE scans of the mouse brain during the process of dying revealed a cascade of pathophysiological events leading to marked brain stiffening within a few minutes after respiratory arrest.^[^ ^139^ ^]^ The results showed that hyperperfusion through vasodilation following hypoxia‐induced acidosis caused extracellular water to be shifted into intracellular, neuronal compartments and resulted in the formation of cytotoxic edema, ultimately leading to postmortem brain stiffening.^[^ ^139^ ^]^ Although overlapping effects prevent a causal interpretation of many in vivo MRE findings, the neural network seems to establish an elastic‐solid scaffold to the brain. **Table** **1** summarizes the in vivo studies mentioned herein.

**Table 1:** Effects of the four interrelated networks of brain mechanics made up of neurons, glial cells, vasculature, and ECM on the coarse‐grained viscoelastic brain properties as discussed in the text. Only in vivo studies are considered here.

Network	Species	Studied effect	Correlation with stiffness	Correlation with tissue fluidity^a)^	References
Neurons	Mouse	Maturation	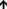 Axonal organization, myelination	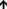 Synaptic plasticity, cellular adhesion (friction)	[111, 113]
Neurons	Cross species	Regional, myelin	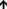 WM vs GM 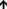 CC vs Hip (mid‐frequency range) 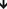 CC vs Hip kHz range	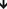 WM vs GM (mid‐frequency range)	[11, 38, 39, 110, 111, 118, 119]
Neurons	Mouse corpus callosum	Demyelination	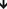	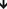	[137]
Neurons	Cross species	White matter fiber bundles	Waveguide, mixed reports ( 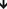 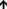 ) w.r.t. fiber direction	N/A	[120, 121, 122, 123]
Neurons	Mouse	Number of neurons	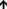 Number of new neurons and neuron density 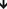 Neural progenitor cells	N/A or 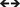	[124, 125, 126, 127
Neurons	Swine, mouse	Postmortem effects	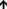 Neuronal swelling	N/A	^[^ ^138^, ^139^ ^]^
Glial cells	Mouse	Maturation	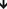 Oligodendrocyte expression profiles	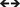	[111]
Glial cells	Mouse	Inflammation	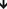 Loss of astrocyte‐vessel connections	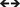	[56]
ECM	Murine hippocampus	Neurogenic niche	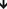 Chondroitin sulfate proteoglycans	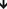	[113, 124]
ECM	Mouse cortex	Sex‐specific ECM differences	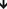 Fibronectin, laminin, and collagen IV 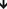 Male vs female	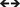	[162, 163]
ECM	Mouse cerebellum	BBB disruption	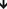 Fibronectin in acute EAE lesions	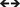	[56]
ECM	Mouse cortex	PNN degradation in EAE	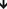 Hyaluronic acid, reduction in chondroitin sulfate	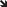	[165]
ECM	Mouse cortex	Sleep‐wake cycle	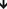 Water content, glymphatic fluid flow	N/A	[187]
Vasculature	Human	Arterial pulsation	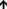 Arterial pulse wave	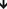 Arterial pulse wave	[194, 199]
Vasculature	Mouse	Cerebral arterial occlusion / stroke	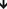 CBF reduction 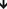 formation of neovessels and 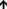 edema	N/A	[25, 201, 206]
Vasculature	Human deep gray matter	Regional CBF differences	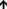 Perfusion pressure	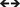	[204]
Vasculature	Human	Dehydration	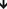 Urine osmolality	N/A	[21]
Vasculature	Human	Hypercapnia	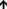 CBF	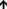 Vessel diameter	[205, 211]
Vasculature	Mouse	Hypothermia	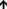 CBF	N/A	[212]
Vasculature	Human	Valsalva maneuver	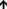	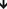	
Vasculature	Human	Lumbar puncture	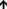 CSF opening pressure	N/A	[213]
Vasculature	Swine	Ventricular pressure	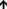 ICP	N/A	[94]
Vasculature	Human, mouse	Functional activation	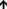 BOLD fMRI signal	N/A	[200, 209, 210]
Emergent patterns					
Tumors	Cross species	Brain tumor aggressiveness	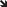 Tumor vs normal tissue 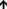 microvessels 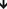 fluid areas	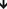 GAG	[170, 171, 173, 174, 175, 179]
Network degradation / remodeling	Cross species	Dementia, gait disorders, NPH, inflammation, temporal lobe epilepsy	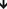 Region‐ and disease‐specific patterns of degradation 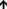 Hippocampal sclerosis	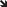 Region‐ and disease‐specific patterns	[134, 218, 219, 221, 227, 228]
Network integrity	Humans	Aging, memory performance, aerobic fitness	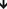 Age 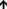 Memory 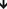 BMI	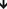 Age 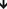 Memory^b)^ 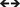 BMI	[80, 106, 114, 115, 116, 128, 131, 236, 237]

^a)^ fluidity measured by the loss angle of the complex shear modulus G* = G’+iG’’: φ = arctan(G’’/G’) or by the powerlaw exponent α of the viscoelastic spring‐pot model: α = 2 φ /π.;^[^ ^13^ ^]^

b) based on the damping ratio ξ = G″/2G′. Abbreviations: BBB – blood‐brain barrier, CBF – cerebral blood flow, PNN – perineuronal nets, GAG – glycosaminoglycans, ICP – intracranial pressure, ECM extracellular matrix, BMI – body mass index, Hip – hippocampus, CC – corpus callosum. Symbols: 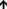 positive correlation, 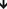 negative correlation, 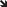 trend toward softer or less fluid properties, 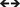 no significant change.

### 5.2 Glial Network

Given that individual glial cells are softer than neurons,^[^ ^140^ ^]^ it is plausible that the accumulation of glial cells in glial scars leads to softer tissue properties, as observed with AFM in rodents.^[^ ^141^ ^]^ In contrast to stiff scars in collagen‐1‐rich tissues, the soft behavior of glial scars is potentially related to glial cell responses associated with demyelination of neuronal axons and disintegrated neural network connections in the injured tissue as well as ECM alterations. Conversely, one can speculate that the proliferation of oligodendrocytes and the formation of glial networks might increase brain stiffness by crosslinking cellular networks and vascular elements, thus reinforcing the brain's mechanical scaffold. This is suggested by studies in mouse brain slices showing that hippocampal and cerebellar stiffness increases with astrocyte number.^[^ ^112^ ^]^ Furthermore, gap junctions coupling astrocytes, oligodendrocytes, and neurons are rich in mechanoresponsive connexins, suggesting that glial networks respond to mechanical forces and actively contribute to brain stiffness.^[^ ^142^ ^]^ This hypothesis is supported by MRE studies in the maturing mouse brain showing that upregulation of proteins expressed by oligodendrocytes correlates with higher stiffness, which, however, may not be decoupled from an increase in neuronal density.^[^ ^111^ ^]^ Conversely, loss of connectivity between networks would lead to softening, similar to observations made in glial scars.^[^ ^141^ ^]^ Some processes involved in neuroinflammation have been shown to be associated with the detachment of glial network elements such as degraded astrocyte‐vessel connections in the rodent brain^[^ ^56^ ^]^ (Figure 3E), which might explain the softening of the brain observed in patients with MS^[^ ^143^, ^144^, ^145^ ^]^ and in experimental autoimmune encephalomyelitis (EAE) mice.^[^ ^146^, ^147^ ^]^ However, the pathophysiology behind neuroinflammation is very complex and involves multiple events that alter ECM structures, as will be discussed in the following section.

### 5.3 Extracellular Matrix

**Figure 6:**
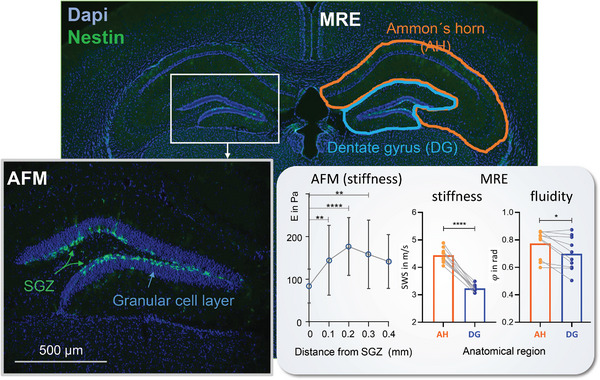
In vivo stiffness of the subregions of the murine hippocampus. Immunofluorescence staining of the murine hippocampus showing nestin, a neuronal progenitor cell marker, in green and cell bodies with DAPI in blue. Atomic force microscopy (AFM) shows softer mechanical properties close to the subgranular zone (SGZ), the neurogenic niche of the hippocampus. Magnetic resonance elastography (MRE) reveals heterogeneity in mechanical properties of the hippocampus, with softer and less fluid properties of the dentate gyrus, comprising the SGZ, compared with Ammon´s horn.^[^ ^124^ ^]^ Note that AFM and MRE were operated at different dynamic regimes and measured different mechanical parameters (Young's modulus E in AFM and shear wave speed SWS / fluidity φ in MRE). In contrast to the stiffness differences between hippocampus and corpus callosum measured by AFM and MRE (Figure 5), both methods provide the same relative changes in mechanical parameters when considering similar tissue regions (*n* = 15 [AFM], *n*  =  10 [MRE], *p  =  0.0102, **p  =  0.0069, ***p  =  0.0005, ****p < 0.0001). Figure adapted with permission from.^[^ ^124^ ^]^

Specific influences of brain ECM on in vivo viscoelastic properties of the CNS cannot be disentangled from cell mechanical properties because mechanosensitive cell surface receptors interact with ECM proteins and mediate cell stiffness through the cytoskeleton.^[^ ^4^, ^148^, ^149^, ^150^ ^]^ Many studies have shown that the mechanical properties of neurons, astrocytes, and microglia are modulated by extracellular mechanics.^[^ ^4^, ^151^, ^152^, ^153^, ^154^ ^]^ In vitro AFM^[^ ^155^ ^]^ and in vivo USE in mice^[^ ^113^ ^]^ have revealed ECM stiffness to regulate the proliferation, differentiation, and aging of CNS progenitor cells.^[^ ^156^, ^157^, ^158^ ^]^ Neurogenesis in the hippocampal dentate gyrus is observed throughout the lifespan of mammals, and stiffness gradients guide the differentiation and migration of CNS progenitor cells.^[^ ^159^, ^160^ ^]^ The regulatory function of ECM stiffness in neurogenesis was demonstrated by enzymatic digestion of chondroitin sulfate proteoglycans – one of the major ECM components in the premature brain.^[^ ^161^ ^]^ The fact that hippocampal stiffness increases during maturation while neurogenesis is progressively downregulated suggests that softness of the neurogenic niche is a fundamental guiding principle of neurogenesis.^[^ ^157^, ^161^ ^]^ Probably for this reason, in vivo MRE of the mouse detects the dentate gyrus as a soft niche with softness that correlates with neurogenic activity (**Figure** **6**).^[^ ^124^ ^]^ Also, sex‐specific ECM differences in basement membrane components were found to correlate with softer properties in the healthy male versus female murine cortex.^[^ ^162^ ^]^ Here, lower expression of laminin and collagen IV but higher expression of fibronectin in male versus female brains correlated with softer properties.^[^ ^162^ ^]^ Similarly, the underlying mechanisms of in vivo brain softening in neuroinflammation seem to be associated with ECM remodeling, as mentioned above, but also with altered endothelial matrix components and local inflammatory processes.^[^ ^57^ ^]^ Specifically, MRE detected softening in areas where accumulation of magnetic nanoparticles administered as MRI contrast agent indicated typical hallmarks of neuroinflammation such as gliosis, leukocyte extravasation, and reduced GAG sulfation.^[^ ^57^ ^]^ Also, the magnitude of tissue remodeling at sites of BBB breakdown, as indicated by the overexpression of fibronectin in acute EAE lesions, correlated with the degree of tissue softening in the mouse cerebellum.^[^ ^56^ ^]^ In this setting, fibronectin reflects the detachment of astrocytic endfeet from blood vessels with weakening of glial‐vascular mechanical crosslinks and, thus, tissue softening. This mechanism might explain in part why the brain softens during the acute phase of EAE and why it stiffens during EAE remission.^[^ ^56^, ^147^ ^]^ This mechanism is a good example for the combined effects of different substructures as it implies that fibronectin (network #3, ECM) is overexpressed as a consequence of astrocytic endfeet (network #2) detachment from blood vessels (network #4). Fibronectin, more than other components of the basement membrane, has been shown to critically influence the viscoelastic response of brain tissue to shear oscillatory forces in *ex vivo* human brain specimens.^[^ ^28^ ^]^ While EAE‐related remodeling of the basement membrane, characterized by increased fibronectin and reduced laminin and collagen IV, mainly reduces brain stiffness,^[^ ^28^, ^162^, ^163^ ^]^ changes in ECM GAGs also influence tissue fluidity. Specifically, reduction in chondroitin sulfate and upregulation of hyaluronic acid (HA) have been found to reduce both brain stiffness and tissue fluidity, probably through the establishment of large networks and reduced polar water ECM interactions (friction), respectively.^[^ ^164^, ^165^, ^166^ ^]^ Importantly, by their amounts and sulfation patterns, chondroitin sulfates can indicate the degradation of PNNs in a way that follows the remitting‐relapsing disease path of EAE.^[^ ^165^ ^]^ This loss of PNN integrity in cortical brain regions is specific to inflammation and is associated with marked tissue softening, which is why it has been proposed as a potential MRE marker of cortical tissue involvement in MS patients.^[^ ^165^ ^]^

ECM alterations also play a critical role in the mechanical behavior of brain tumors in terms of aggressiveness and invasive growth.^[^ ^107^, ^167^ ^]^ AFM‐based studies of patient tissue showed that glioma aggressiveness, from gliosis to low‐grade gliomas to grade IV gliomas, progressively increased with HA and tenascin‐C expression levels, which in turn correlated with ECM stiffness.^[^ ^168^ ^]^ This may explain the progressive tumor softening shown by in vivo MRE during radiation therapy in a murine model of glioblastoma.^[^ ^169^ ^]^ However, an inverse correlation between tumor aggressiveness and tumor stiffness was suggested by MRE in glioma, showing that tumors with isocitrate dehydrogenase 1 (IDH1) mutation were significantly stiffer than those with the wild‐type form of the IDH1 gene – a diagnostic marker for poorer prognosis.^[^ ^170^ ^]^ The establishment of clinical thresholds for a stiffness‐based classification of brain tumors is hampered by significant intratumoral heterogeneity. Areas of higher cellularity and perfused (pressurized) microvessels coincide with higher stiffness values while necrotic and fluid areas have lower stiffness.^[^ ^171^ ^]^ As heterogeneity increases with tumor progression, it has been proposed to leverage stiffness heterogeneity as a clinical MRE tumor marker.^[^ ^172^ ^]^ Tumor heterogeneity is also the reason why average stiffness values reported in the MRE literature vary largely and there is only a tendency toward lower values than found in normal‐appearing tissue with large overlap between entities.^[^ ^173^, ^174^ ^]^ Tissue fluidity seems to have a higher discriminative power between benign and malignant neoplasms than stiffness. Fluidity is reduced in glioblastomas compared with other, less invasive, brain tumors, suggesting that it has a critical role in a tumor's infiltrative behavior.^[^ ^175^ ^]^ It has been speculated that the excess of sulfated GAGs in glioblastomas^[^ ^176^ ^]^ may contribute to gel‐like confined water molecules and overall soft‐solid material behavior that promotes invasive and irregular boundaries^[^ ^177^ ^]^ according to the viscous fingering theory.^[^ ^175^ ^]^ This theory states that, in an expanding medium, which has lower viscosity and surface tension than its environment, viscous fingers form and penetrate the surrounding matrix.^[^ ^178^ ^]^ In this simplified, physics‐based picture, brain tumors with soft‐elastic (low fluidity) behavior grow infiltratively while stiff‐viscous properties (high fluidity) favor displacing tumor expansion. This pattern has been observed in patients and agrees with results obtained in a xenograft mouse model, in which angiogenic treatment attenuated the decrease in stiffness and fluidity of glioblastomas.^[^ ^179^ ^]^ However, the observed treatment effects could have resulted from a variety of overlapping mechanisms, including ECM changes, vascular normalization, myelin preservation, and changes in cell motility.^[^ ^179^ ^]^ Particularly in tumors, tissue fluidity appears to be sensitive to jamming‐unjamming transitions,^[^ ^180^ ^]^ which translate single‐cell mechanical properties into the macroscopic contrast of in vivo MRE through emergent multicellular behavior.^[^ ^32^, ^181^ ^]^

Along with CSF spaces and ISF, the ECM contributes to the poroelastic properties of the brain.^[^ ^182^, ^183^ ^]^ As discussed above, GAGs bind large amounts of water^[^ ^184^ ^]^ and are important for the elastic‐solid tissue response by reducing viscosity in nonpolar, unsulfated GAGs such as HA while increasing viscosity / fluidity through polar ECM‐water interactions.^[^ ^166^ ^]^ Unlike water retained within ECM networks through the interactions with polar GAGs,^[^ ^166^ ^]^ water in pores such as those formed by the glymphatic system^[^ ^185^ ^]^ can move relatively freely, giving rise to long MRI relaxation times on the order of CSF relaxation times. The differences in T1 relaxation times between CSF and parenchyma have been used to quantify porosity in the human brain, which is approximately 14% in GM and 5% in WM.^[^ ^186^ ^]^ Optical coherence elastography revealed marked softening of the mouse cortex during anesthesia‐induced sleep, likely related to changes in glymphatic extracellular fluid channels during the sleep‐wake cycle.^[^ ^187^ ^]^ In contrast, tumor‐related increases in ECM water content, as manifest in perifocal edema, slightly increase the shear stiffness of human brain matter^[^ ^175^ ^]^ while edema developing in response to brain injury in the mouse was reported to soften brain tissue.^[^ ^188^ ^]^ The disparate behavior of viscoelastic properties in brain edematous tissue might be related to the time of injury. While edema and tissue necrosis that occur early after contusion make the brain softer and less viscous, later changes including reactive astroglial alterations tend to make the brain stiffer than normal.^[^ ^189^ ^]^ Also, the tissue pressure exerted by a growing mass might play a role in tissue stiffening^[^ ^179^ ^]^ due to compressive strain^[^ ^190^ ^]^ whereas subsequent softening could be the result of degraded neural networks and increased extracellular water content.^[^ ^91^, ^191^ ^]^ The effects of lesion‐induced edema can be detected even in the ipsilateral hemisphere because static pressure and compression affect large areas of the brain in a virtually undamped fashion.^[^ ^25^, ^179^ ^]^

### 5.4 Vasculature

The brain is a tightly perfusion‐regulated, pulsating organ.^[^ ^192^ ^]^ With each heartbeat, an ICP pulse wave is generated when the ascending arterial pulse from the heart arrives at the midbrain through the large cerebral arteries.^[^ ^193^ ^]^ That pressure wave traverses brain hemispheres within ≈2 ms (50 ms^−1^ compression wave speed at 50 Hz as estimated in^[^ ^194^ ^]^). Thus, compression wave speed in the brain is much slower than in free water (1500 ms^−1^) because it is decelerated by poroelastic fluid‐solid interactions. Furthermore, the ability of the brain to expand in volume is highly dependent on the rate of deformation. During arterial pulsation, brain volume expands by ≈0.5 mL^[^ ^195^ ^]^ and by up to 3 mL during a static increase in ICP induced by the Valsalva maneuver.^[^ ^196^ ^]^

The arterial pulse wave is a shear wave spreading throughout the brain along the vascular tree from the arteries into the capillary bed, which drives pulsatile fluid flow in the brain. Unlike a compression wave, the arterial pulse wave is a vascular distensibility wave whose amplitude and propagation speed depend on the shear modulus of the vessel walls. As a result, this vascular pulse wave travels at slower velocities from 0.4 ms^−1^ (in proximal segments of the middle cerebral artery^[^ ^197^ ^]^) down to only 0.3 mms^−1^ in the neocortical capillaries of the mouse brain.^[^ ^198^ ^]^ The passage of the fluid pulse wave during cranial systole is associated with a transient increase in the brain's fluid fraction, which could explain why the brain becomes softer and more fluid‐like during cranial systole as measured by MRE.^[^ ^194^ ^]^ However, tissue softening due to increased fluid content (fluid fraction) depends on compliant tissue expansion, i.e., expansion without the generation of stresses that may cause compression stiffening or hyperelastic vascular stiffening. Such a scenario of compliant vascular expansion is unlikely to occur during the passage of the pressure‐driven arterial pulse wave in the brain. Probably for this reason, a recent in vivo USE study with higher temporal resolution than MRE found that brain tissue stiffens in synchrony with cerebral arterial pulsation.^[^ ^199^ ^]^ Conversely, softening due to higher fluid content is consistent with contemporary reports of a reduction in brain stiffness upon functional activation^[^ ^200^ ^]^ that correlates with tissue water^[^ ^187^ ^]^ as well as with predictions of the stress relaxation model^[^ ^89^ ^]^ of decreased shear stress after vasodilation. The increase in fluid volume might also explain the observation that mouse brain softened after middle cerebral arterial occlusion and formation of neovessels.^[^ ^201^ ^]^ However, beyond fluid volume, there are several other perfusion‐related effects that potentially influence effective‐medium brain properties. The following, partially counteracting, effects resulting from changes in vessel diameter need to be considered:

1) Fluid fraction changes (e.g., stiffness is reduced by compliant expansion and increase in fluid content).^[^ ^187^, ^194^, ^200^ ^]^
2) Vascular wall stiffening through hyperelastic expansion or smooth muscle contraction causing an increase in brain stiffness^[^ ^21^, ^24^, ^202^, ^203^ ^]^ or softening upon relaxation.^[^ ^21^ ^]^
3) Regional effects on perfusion pressure due to differences in vascular anatomy or aberrant tumor vessels (smaller vessels with higher perfusion pressure are associated with higher stiffness).^[^ ^204^ ^]^
4) Blood viscosity changes (blood viscosity increases with vasodilation through the Fåhraeus‐Lindqvist effect).^[^ ^205^ ^]^

Consequently, different effects of CBF on brain viscoelasticity values have been reported (see references in the list above). As a baseline, regional differences in CBF‐stiffness interactions were observed in deep GM of healthy subjects (#3 in the list above).^[^ ^204^ ^]^ In these subjects, stiffness correlated with CBF normalized by vascular area (A), which is proportional to the flux rate or perfusion pressure gradient. Of note, CBF/A is higher in the striatum than in the hippocampus, thalamus, or globus pallidus and is associated with higher stiffness values in that region. The markedly higher stiffness of the putamen and nucleus accumbens compared with other deep GM regions may indicate the sensitivity of brain stiffness to perfusion pressure and thus may also correlate with the known vulnerability of striatal regions to stroke.^[^ ^204^ ^]^ In contrast to pressure‐driven stiffness increases, the formation of neovessels in neurotumors^[^ ^206^ ^]^ causes a decline of stiffness, as observed in stroke,^[^ ^207^ ^]^ probably due to a compliant, stress‐free, increase in the fluid tissue fraction while anti‐angiogenic treatment has been found to positively influence stiffness and fluidity in glioblastoma.^[^ ^208^ ^]^

In general, CBF is autoregulated within a narrow range according to the brain tissue's demand for nutrients and oxygen, as under functional activation,^[^ ^200^, ^209^, ^210^ ^]^ hypoxia,^[^ ^139^ ^]^ or hypercapnia.^[^ ^205^, ^211^ ^]^ Physiological changes in CBF were observed in response to the Valsalva maneuver,^[^ ^24^, ^203^ ^]^ low body temperature,^[^ ^212^ ^]^ and dehydration.^[^ ^21^ ^]^ All of these different conditions have been studied with MRE and USE, and the results consistently suggest that increased blood perfusion is associated with stiffening of brain tissue. Remarkably, all neurological conditions associated with a higher ICP have been reported as states of elevated brain stiffness, motivating the use of brain stiffness as a noninvasive marker of ICP.^[^ ^94^ ^]^ First encouraging studies were performed using USE in a mouse model of ischemic stroke^[^ ^25^ ^]^ and in patients with idiopathic intracranial hypertension before and after lumbar puncture.^[^ ^213^ ^]^ In patients, abnormally high stiffness values dropped after brain decompression with excellent diagnostic accuracy.^[^ ^213^ ^]^ In addition to stiffness, viscosity seems to be sensitive to cerebral blood perfusion. Studies indicate that viscosity is affected by changes in vascular diameter. While dilation of vessels due to arterial pulsation or hypercapnia led to increased fluidity,^[^ ^194^, ^205^ ^]^ constriction of cerebral vessels, particularly in the venous bed, during the Valsalva maneuver (hindering the outflow of blood from the brain) led to significantly lower viscosity in humans.^[^ ^203^ ^]^ A possible explanation is provided by the Fåhraeus‐Lindqvist effect, which states that blood viscosity increases upon vascular dilation. Blood viscosity is minimal in vessels with diameters ≈4–5 µm.^[^ ^214^ ^]^ In this relatively simple explanation, viscosity changes mirror the apparent viscosity of blood and its relationship to vessel size. Finally, it should be noted that the mechanical vibrations induced in the brain at the frequency range used in MRE modulate CBF and blood pressure, which in turn may affect the measured stiffness values.^[^ ^215^, ^216^ ^]^ It seems that, even in the brain, there is no observation without interaction.

## 6 Emergent Patterns

As noted above, in vivo brain viscoelastic properties on the macroscopic scale are the result of many overlapping interactions and should be considered as effective‐medium properties reflecting the emergence of all four networks and their components discussed in this article. Therefore, many studies in the literature report interesting and diagnostically relevant mechanical property changes, which, however, cannot be attributed to specific mechanisms or still have unknown origins within the hierarchy of viscoelastic elements in the brain. Given our hypothesis of the four intertwined mechanical networks that make up the brain's scaffold, any weakening of one or more of these elements –neuronal, glial, or vascular dysfunction – as well as alterations of the ECM including loss of connectivity between these networks would ultimately lead to softening of brain tissue on a coarse‐grained scale. Consequently, almost all brain pathologies studied so far using MRE showed brain softening, including MS,^[^ ^143^, ^144^, ^145^, ^217^ ^]^ neuromyelitis optica,^[^ ^218^ ^]^ Parkinson's disease,^[^ ^219^, ^220^ ^]^ and Alzheimer's disease^[^ ^133^, ^134^, ^135^ ^]^ while stiffening was only reported for the hippocampus of patients with temporal lobe epilepsy.^[^ ^221^ ^]^ Interestingly, as in most brain diseases, when stiffness decreased, tissue fluidity was also reduced.^[^ ^106^, ^222^, ^223^, ^224^, ^225^, ^226^, ^227^ ^]^ Disparate results have been reported for pressure‐related conditions such as normal pressure hydrocephalus^[^ ^227^, ^228^, ^229^, ^230^, ^231^, ^232^, ^233^ ^]^ and intracranial hypertension,^[^ ^213^, ^234^ ^]^ for which a tendency toward tissue stiffening was observed as long as ICP was elevated^[^ ^213^, ^234^ ^]^ while softening was found at the stage of chronic pressure‐related tissue degeneration.^[^ ^235^ ^]^ Different regional patterns of softening have been identified and shown to correlate with the degree of tissue involvement in dementia, gait disorders, and inflammation.^[^ ^134^, ^218^, ^219^, ^228^ ^]^ These studies, along with reports of physiological softening with age,^[^ ^80^, ^128^, ^131^, ^236^ ^]^ softer properties in association with reduced memory performance^[^ ^237^ ^]^ as well as softer striatal reward systems in overweight individuals,^[^ ^238^ ^]^ provide overwhelming evidence that reduced stiffness is a common signature of impaired brain tissue integrity. Exceptions are hippocampal sclerosis,^[^ ^221^ ^]^ elevated ICP,^[^ ^213^ ^]^ and tumors.^[^ ^173^, ^174^, ^239^, ^240^ ^]^

**Figure 7:**
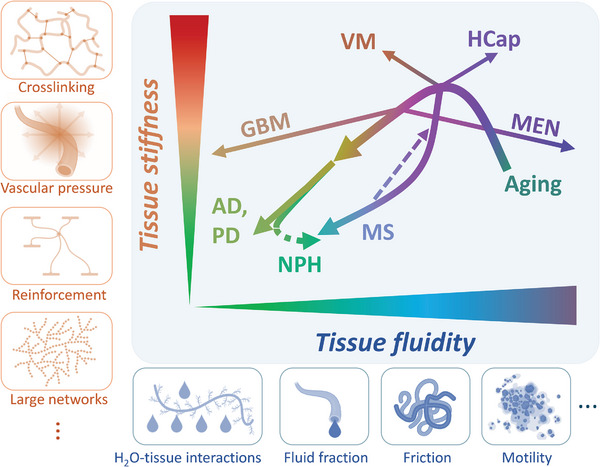
In vivo changes in brain stiffness and tissue fluidity observed in published studies using in vivo MRE. Brain development has been shown to be associated with an increase in stiffness and a decrease in fluidity, leading to more pronounced elastic‐solid properties,^[^ ^111^ ^]^ which, after the maturation phase, progress toward softening due to aging.^[^ ^241^ ^]^ Multiple sclerosis (MS),^[^ ^222^ ^]^ Alzheimer's disease (AD),^[^ ^242^ ^]^ Parkinson's disease (PD),^[^ ^225^ ^]^ and normal pressure hydrocephalus (NPH)^[^ ^224^ ^]^ are all associated with local softening and a shift in brain tissue fluidity toward elastic‐solid behavior. Treatment effects (dashed lines) in NPH have been shown to restore fluidity,^[^ ^235^ ^]^ whereas in the mouse model of MS, disease remission restored stiffness and fluidity, highlighting the transient nature of MS‐related mechanical tissue remodeling.^[^ ^165^ ^]^ In contrast to other diseases and tumors outside the CNS, brain tumors such as glioblastoma (GBM) and meningioma (MEN) primarily affect tissue fluidity with only marginal changes in stiffness toward softer properties.^[^ ^31^ ^]^ Changes in blood flow and pressure, resulting, for example, from the Valsalva maneuver (VM)^[^ ^203^ ^]^ or inhalation of CO_2_‐enriched air (hypercapnia, HCap),^[^ ^205^ ^]^ cause brain stiffening, but with variable effects on tissue fluidity, which in turn depend on the changes induced in vessel diameter, perfusion pressure, and fluid fraction. Based on the studies shown here and discussed in the text, we have identified emergent patterns of tissue architectural changes that lead to the observed characteristic changes within the 2D stiffness‐fluidity space as illustrated by the cartoons in orange for stiffness and blue for fluidity.

Table 1 summarizes the discussed effects of the four brain networks on in vivo mechanical parameters reported in the literature. **Figure** **7** shows emergent patterns of brain stiffness and tissue fluidity as detected by in vivo MRE. Based on the results summarized in Table 1 and the discussion in the text, we identified four basic physical processes that affect brain stiffness and tissue fluidity. As illustrated in Figure 7, large networks, fiber reinforcement, crosslinking, and vascular pressure, have been identified as the physical interactions that support stiffness, independent of the contributors to tissue fluidity, which are polar tissue‐water interactions, fluid fraction, friction, and cell motility.

## 7 Summary and Conclusion

This article provides an overview of the different structural components of the brain and their contributions to the mechanical parameters that are measured by MRE. The brain contains two major cellular networks, the neuronal and glial networks, which cooperate and support each other's functions. The extracellular compartment of the brain consists of ECM and extracellular fluids, including ISF, CSF, and blood. All of these components and their distribution collectively shape the brain's coarse‐grained mechanical properties, including stiffness and viscosity, as measured by MRE. Consequently, MRE has been proven sensitive to pathophysiological processes that affect the mechanical concert of the four basic interconnected networks in the brain: neurons, glial cells, ECM, and vasculature. Tissue alterations due to neuronal degeneration, demyelination, inflammation, or vascular leakage have been associated with tissue softening. In contrast, neuronal proliferation, cellular network formation, accumulation of certain matrix components, and higher vascular pressure have been shown to result in brain stiffening. In addition, brain viscosity has been reported to change with blood perfusion, tumor invasion, and brain maturation. All of these processes are highly relevant to the detection and assessment of neurological disorders, suggesting that elastography bears great potential for improving the detection and monitoring of neurological diseases.

## Conflict of Interest

The authors declare no conflict of interest.

## Supporting information

Supporting Information

## References

[1] C. L. Johnson , E. H. Telzer , Dev. Cogn. Neurosci. 2018, 33, 176. 29239832 10.1016/j.dcn.2017.08.010 PMC5832528

[2] P. K. Viji Babu , M. Radmacher , Front. Neurosci. 2019, 13, 600. 31258462 10.3389/fnins.2019.00600 PMC6587663

[3] J. M. Barnes , L. Przybyla , V. M. Weaver , J. Cell Sci. 2017, 130, 71. 28043968 10.1242/jcs.191742 PMC5394781

[4] W. J. Tyler , Nat. Rev. Neurosci. 2012, 13, 867. 23165263 10.1038/nrn3383

[5] M. Kaiser , C. C. Hilgetag , Front. Neuroinform. 2010, 4, 713. 10.3389/fninf.2010.00008 PMC2876872 20514144

[6] U. Lee , G. Oh , S. Kim , G. Noh , B. Choi , G. A. Mashour , J. Am. Soc. Anesthesiol. 2010, 113, 1081. 10.1097/ALN.0b013e3181f229b5 PMC2962769 20881595

[7] D. Meunier , R. Lambiotte , E. T. Bullmore , Front. Neurosci. 2010, 4, 200. 21151783 10.3389/fnins.2010.00200 PMC3000003

[8] T. I. Józsa , R. M. Padmos , N. Samuels , W. El‐Bouri , A. G. Hoekstra , S. J. Payne , Interface Focus 2021, 11, 20190127. 33343874 10.1098/rsfs.2019.0127 PMC7739914

[9] J. Faber , J. Hinrichsen , A. Greiner , N. Reiter , S. Budday , Curr. Protoc. 2022, 2, e381. 35384412 10.1002/cpz1.381

[10] S. Budday , T. C. Ovaert , G. A. Holzapfel , P. Steinmann , E. Kuhl , Arch. Comput. Methods Eng. 2020, 27, 1187.

[11] J. Guo , S. Hirsch , A. Fehlner , S. Papazoglou , M. Scheel , J. Braun , I. Sack , PLoS One 2013, 8, e71807. 23977148 10.1371/journal.pone.0071807 PMC3743755

[12] I. Sack , B. Beierbach , U. Hamhaber , D. Klatt , J. Braun , NMR Biomed. 2008, 21, 265. 17614101 10.1002/nbm.1189

[13] I. Sack , Nat. Rev. Phys. 2023, 5, 25.

[14] Z. Yin , A. J. Romano , A. Manduca , R. L. Ehman , J. Huston 3rd , Top. Magn. Reson. Imaging 2018, 27, 305. 30289827 10.1097/RMR.0000000000000178 PMC6176744

[15] M. C. Murphy , J. Huston 3rd , R. L. Ehman , NeuroImage 2019, 187, 176. 28993232 10.1016/j.neuroimage.2017.10.008 PMC5889749

[16] M. Nanjappa , A. Kolipaka , Magn. Reson. Imaging Clinics 2021, 29, 617. 10.1016/j.mric.2021.06.011 34717849

[17] A. M. Khair , G. McIlvain , M. D. J. McGarry , V. Kandula , X. Yue , G. Kaur , L. W. Averill , A. K. Choudhary , C. L. Johnson , R. M. Nikam , Pediatr. Radiol. 2023, 53, 2712. 37794174 10.1007/s00247-023-05779-3 PMC11086054

[18] Y. Feng , M. C. Murphy , E. Hojo , F. Li , N. Roberts , J. Magn. Reson. Imaging 2024, 59, 82. 37084171 10.1002/jmri.28747

[19] L. V. Hiscox , C. L. Johnson , E. Barnhill , M. D. McGarry , J. Huston , E. J. van Beek , J. M. Starr , N. Roberts , Phys. Med. Biol. 2016, 61, R401. 27845941 10.1088/0031-9155/61/24/R401

[20] H. G. Kim , M. S. Park , J. D. Lee , S. Y. Park , J. Ultrasound. Med. 2017, 36, 1313. 28304105 10.7863/ultra.16.06079

[21] B. Kreft , J. Bergs , M. Shahryari , L. A. Danyel , S. Hetzer , J. Braun , I. Sack , H. Tzschätzsch , Front. Physiol. 2020, 11, 616984. 33505319 10.3389/fphys.2020.616984 PMC7830390

[22] Y.‐L. Liu , D. Liu , L. Xu , C. Su , G.‐Y. Li , L.‐X. Qian , Y. Cao , J. Mech. Behav. Biomed. Mater. 2018, 83, 120. 29702328 10.1016/j.jmbbm.2018.04.017

[23] T. Selbekk , R. Brekken , O. Solheim , S. Lydersen , T. A. Hernes , G. Unsgaard , Ultrasound Med. Biol. 2010, 36, 2. 19854562 10.1016/j.ultrasmedbio.2009.05.007

[24] H. Tzschatzsch , B. Kreft , F. Schrank , J. Bergs , J. Braun , I. Sack , Sci. Rep. 2018, 8, 17888. 30559367 10.1038/s41598-018-36191-9 PMC6297160

[25] Z. S. Xu , R. J. Lee , S. S. Chu , A. Yao , M. K. Paun , S. P. Murphy , P. D. Mourad , J. Ultrasound in Med. 2013, 32, 485. 23443189 10.7863/jum.2013.32.3.485

[26] R. M. S. Sigrist , J. Liau , A. E. Kaffas , M. C. Chammas , J. K. Willmann , Theranostics 2017, 7, 1303. 28435467 10.7150/thno.18650 PMC5399595

[27] C. M. Hall , E. Moeendarbary , G. K. Sheridan , Eur. J. Neurosci. 2021, 53, 3851. 32356339 10.1111/ejn.14766

[28] K. Linka , N. Reiter , J. Wurges , M. Schicht , L. Brauer , C. J. Cyron , F. Paulsen , S. Budday , Front. Bioeng. Biotechnol. 2021, 9, 704738. 34485258 10.3389/fbioe.2021.704738 PMC8415910

[29] L. V. Hiscox , H. Schwarb , M. D. J. McGarry , C. L. Johnson , Neuroimage 2021, 232, 117889. 33617995 10.1016/j.neuroimage.2021.117889 PMC8251510

[30] E. K. Pillai , K. Franze , Neuron 2024, 112, 342. 37967561 10.1016/j.neuron.2023.10.005

[31] K.‐J. Streitberger , L. Lilaj , F. Schrank , J. Braun , K.‐T. Hoffmann , M. Reiss‐Zimmermann , J. A. Käs , I. Sack , Proc. Natl. Acad. Sci. USA 2020, 117, 128. 31843897 10.1073/pnas.1913511116 PMC6955323

[32] F. Sauer , S. Grosser , M. Shahryari , A. Hayn , J. Guo , J. Braun , S. Briest , B. Wolf , B. Aktas , L.‐C. Horn , I. Sack , J. A. Käs , Adv. Sci. (Weinh) 2023, 10, e2303523. 37553780 10.1002/advs.202303523 PMC10502644

[33] I. Molina‐Gonzalez , R. K. Holloway , Z. Jiwaji , O. Dando , S. A. Kent , K. Emelianova , A. F. Lloyd , L. H. Forbes , A. Mahmood , T. Skripuletz , V. Gudi , J. A. Febery , J. A. Johnson , J. H. Fowler , T. Kuhlmann , A. Williams , S. Chandran , M. Stangel , A. J. M. Howden , G. E. Hardingham , V. E. Miron , Nat. Commun. 2023, 14, 3372. 37291151 10.1038/s41467-023-39046-8 PMC10250470

[34] F. A. C. Azevedo , L. R. B. Carvalho , L. T. Grinberg , J. M. Farfel , R. E. L. Ferretti , R. E. P. Leite , W. J. Filho , R. Lent , S. Herculano‐Houzel , J. Comp. Neurol. 2009, 513, 532. 19226510 10.1002/cne.21974

[35] S. Herculano‐Houzel , B. Mota , R. Lent , Proc. Natl. Acad. Sci. USA 2006, 103, 12138. 16880386 10.1073/pnas.0604911103 PMC1567708

[36] S. Herculano‐Houzel , Front. Hum. Neurosci 2009, 3, 31. 19915731 10.3389/neuro.09.031.2009 PMC2776484

[37] C. L. Call , D. E. Bergles , Nature Comm. 2021, 12, 4767. 10.1038/s41467-021-25035-2 PMC8346564 34362912

[38] J. Weickenmeier , R. de Rooij , S. Budday , T. C. Ovaert , E. Kuhl , J. Mech. Behav. Biomed. Mater. 2017, 76, 119. 28462864 10.1016/j.jmbbm.2017.04.017

[39] J. Weickenmeier , R. de Rooij , S. Budday , P. Steinmann , T. C. Ovaert , E. Kuhl , Acta Biomater. 2016, 42, 265. 27475531 10.1016/j.actbio.2016.07.040

[40] K. Franze , P. A. Janmey , J. Guck , Annu. Rev. Biomed. Eng. 2013, 15, 227. 23642242 10.1146/annurev-bioeng-071811-150045

[41] C. Sampaio‐Baptista , H. Johansen‐Berg , Neuron 2017, 96, 1239. 29268094 10.1016/j.neuron.2017.11.026 PMC5766826

[42] R. G. Almeida , D. A. Lyons , J. Neurosci. 2017, 37, 10023. 29046438 10.1523/JNEUROSCI.3185-16.2017 PMC6596541

[43] K. A. Southam , A. J. Vincent , D. H. Small , J. Alzheimers. Dis. 2016, 51, 657. 26890782 10.3233/JAD-151075 PMC4927862

[44] L. F. Agnati , M. Marcoli , G. Maura , A. Woods , D. Guidolin , J. Neural. Transm. 2018, 125, 883. 29427068 10.1007/s00702-018-1855-7

[45] M. V. Sofroniew , Nat. Rev. Neurosci. 2015, 16, 249. 25891508 10.1038/nrn3898 PMC5253239

[46] A. Almad , N. J. Maragakis , Nat. Rev. Neurol. 2018, 14, 351. 29769699 10.1038/s41582-018-0010-2

[47] M. Bradl , H. Lassmann , Acta Neuropathol. 2010, 119, 37. 19847447 10.1007/s00401-009-0601-5 PMC2799635

[48] J. F. Ghersi‐Egea , N. Strazielle , M. Catala , V. Silva‐Vargas , F. Doetsch , B. Engelhardt , Acta Neuropathol. 2018, 135, 337. 29368213 10.1007/s00401-018-1807-1

[49] N. M. O'Brown , S. J. Pfau , C. Gu , Genes Dev. 2018, 32, 466. 29692355 10.1101/gad.309823.117 PMC5959231

[50] T. S. Reese , M. J. Karnovsky , J. Cell Biol. 1967, 34, 207. 6033532 10.1083/jcb.34.1.207 PMC2107213

[51] I. Wilhelm , A. Nyul‐Toth , M. Suciu , A. Hermenean , I. A. Krizbai , Tissue Barriers 2016, 4, e1143544. 27141424 10.1080/21688370.2016.1143544 PMC4836475

[52] D. Purves , S. M. Williams , Neuroscience, 2nd ed., Sinauer Associates, Sunderland, MA, USA 2001.

[53] E. Kandel , J. Schwartz , S. S. Jessell , A. J. Hudspeth , Principles of Neural Science, Fifth Edition, McGraw‐Hill Publishing, New York 2012.

[54] C. E. Collins , D. C. Airey , N. A. Young , D. B. Leitch , J. H. Kaas , Proc. Natl. Acad. Sci. USA 2010, 107, 15927. 20798050 10.1073/pnas.1010356107 PMC2936588

[55] B. Fischl , A. M. Dale , Proc. Natl. Acad. Sci. USA 2000, 97, 11050. 10984517 10.1073/pnas.200033797 PMC27146

[56] S. Wang , J. M. Millward , L. Hanke‐Vela , B. Malla , K. Pilch , A. Gil‐Infante , S. Waiczies , S. Mueller , P. Boehm‐Sturm , J. Guo , I. Sack , C. Infante‐Duarte , Front. Neurol. 2019, 10, 1382. 31998225 10.3389/fneur.2019.01382 PMC6970413

[57] R. V. Silva , A. S. Morr , S. Mueller , S. P. Koch , P. Boehm‐Sturm , Y. Rodriguez‐Sillke , D. Kunkel , H. Tzschätzsch , A. A. Kühl , J. Schnorr , M. Taupitz , I. Sack , C. Infante‐Duarte , Front. Neurosci. 2021, 15, 701308. 34497486 10.3389/fnins.2021.701308 PMC8419310

[58] E. Sykova , C. Nicholson , Physiol. Rev. 2008, 88, 1277. 18923183 10.1152/physrev.00027.2007 PMC2785730

[59] S. Hirsch , J. Braun , I. Sack , Viscoelastic Theory. Magnetic Resonance Elastography, Wiley‐VCH Verlag GmbH & Co. KGaA, Weinheim 2016, pp. 61‐129.

[60] A. K. Shetty , G. Zanirati , Aging. Dis. 2020, 11, 462. 32010493 10.14336/AD.2020.0103 PMC6961771

[61] Y. Lei , H. Han , F. Yuan , A. Javeed , Y. Zhao , Prog. Neurobiol. 2017, 157, 230. 26837044 10.1016/j.pneurobio.2015.12.007

[62] E. Burnside , E. Bradbury , Neuropathol. Appl. Neurobiol. 2014, 40, 26. 24438519 10.1111/nan.12114

[63] D. R. Zimmermann , M. T. Dours‐Zimmermann , Histochem. Cell Biol. 2008, 130, 635. 18696101 10.1007/s00418-008-0485-9

[64] J. C. Kwok , G. Dick , D. Wang , J. W. Fawcett , Dev. Neurobiol. 2011, 71, 1073. 21898855 10.1002/dneu.20974

[65] W. Härtig , A. Derouiche , K. Welt , K. Brauer , J. Grosche , M. Mäder , A. Reichenbach , G. Brückner , Brain Res. 1999, 842, 15. 10526091 10.1016/s0006-8993(99)01784-9

[66] W. Härtig , A. Singer , J. Grosche , K. Brauer , O. P. Ottersen , G. Brückner , Brain Res. 2001, 899, 123. 11311873 10.1016/s0006-8993(01)02211-9

[67] S. Hirsch , J. Reichold , M. Schneider , G. Szekelyi , B. Weber , J. Cereb. Blood Flow Metab. 2012, 32, 952. 22472613 10.1038/jcbfm.2012.39 PMC3367227

[68] O. Heubner , Cenralblatt für die medicinischen Wissenschaften 1872, 52, 817.

[69] D. F. Rolfe , G. C. Brown , Physiol. Rev. 1997, 77, 731. 9234964 10.1152/physrev.1997.77.3.731

[70] M. Tata , C. Ruhrberg , A. Fantin , Mech. Dev. 2015, 138, 26. 26222953 10.1016/j.mod.2015.07.001 PMC4678116

[71] J. Hua , P. Liu , T. Kim , M. Donahue , S. Rane , J. J Chen , Q. Qin , S.‐G. Kim , Neuroimage. 2018, 187, 17. 29458187 10.1016/j.neuroimage.2018.02.027 PMC6095829

[72] M. Cavaglia , S. M. Dombrowski , J. Drazba , A. Vasanji , P. M. Bokesch , D. Janigro , Brain Res. 2001, 910, 81. 11489257 10.1016/s0006-8993(01)02637-3

[73] B. A. MacVicar , E. A. Newman , Cold Spring Harb. Perspect. Biol. 2015, 7, 020388. 10.1101/cshperspect.a020388 PMC4448617 25818565

[74] N. A. Jessen , A. S. Munk , I. Lundgaard , M. Nedergaard , Neurochem. Res. 2015, 40, 2583. 25947369 10.1007/s11064-015-1581-6 PMC4636982

[75] N. I. Bower , B. M. Hogan , J. Mol. Med. (Berl) 2018, 96, 383. 29610928 10.1007/s00109-018-1634-9

[76] J. M. Wardlaw , H. Benveniste , M. Nedergaard , B. V. Zlokovic , H. Mestre , H. Lee , F. N. Doubal , R. Brown , J. Ramirez , B. J. MacIntosh , A. Tannenbaum , L. Ballerini , R. L. Rungta , D. Boido , M. Sweeney , A. Montagne , S. Charpak , A. Joutel , K. J. Smith , S. E. Black , Nat. Rev. Neurol. 2020, 16, 137. 32094487 10.1038/s41582-020-0312-z

[77] J. Ormachea , K. J. Parker , Phys. Med. Biol. 2020, 65, 24TR06. 10.1088/1361-6560/abca00 33181486

[78] A. Manduca , P. V. Bayly , R. L. Ehman , A. Kolipaka , T. J. Royston , I. Sack , R. Sinkus , B. E. Van Beers , Magn. Reson. Med. 2021, 85, 2377. 33296103 10.1002/mrm.28627 PMC8495610

[79] H. Tzschätzsch , J. Guo , F. Dittmann , S. Hirsch , E. Barnhill , K. Jöhrens , J. Braun , I. Sack , Med. Image. Anal. 2016, 30, 1. 26845371 10.1016/j.media.2016.01.001

[80] H. Herthum , S. Hetzer , B. Kreft , H. Tzschätzsch , M. Shahryari , T. Meyer , S. Görner , H. Neubauer , J. Guo , J. Braun , I. Sack , Front. Bioeng. Biotechnol. 2022, 10, 1056131. 36532573 10.3389/fbioe.2022.1056131 PMC9755504

[81] Y. Fung , Biomechanics, mechanical properties of living tissue, Springer‐Verlag, Berlin 1993.

[82] S. Holm , Waves with Power‐Law Attenuation, ASA Press /Springer / Springer International Publishing / Springer, Berlin, 2019.

[83] H. S. A. Biphasic , In Quantification of Biophysical Parameters in Medical Imaging (Eds.: Sack I , Schaeffter T ), 1st ed. chap 4 Springer, Berlin 2018, 71.

[84] S. Hirsch , F. Beyer , J. Guo , S. Papazoglou , H. Tzschaetzsch , J. Braun , I. Sack , Phys. Med. Biol. 2013, 58, 5287. 23852144 10.1088/0031-9155/58/15/5287

[85] P. R. Perrinez , F. E. Kennedy , E. E. Van Houten , J. B. Weaver , K. D. Paulsen , IEEE Trans. Biomed. Eng. 2009, 56, 598. 19272864 10.1109/TBME.2008.2009928 PMC2857336

[86] M. D. J. McGarry , C. L. Johnson , B. P. Sutton , J. G. Georgiadis , E. E. W. Van Houten , A. J. Pattison , J. B. Weaver , K. D. Paulsen , Med. Phys. 2015, 42, 947. 25652507 10.1118/1.4905048 PMC4312344

[87] D. Chou , J. C. Vardakis , L. Guo , B. J. Tully , Y. Ventikos , J. Biomech. 2016, 49, 2306. 26671218 10.1016/j.jbiomech.2015.11.025

[88] H. Herthum , S. C. H. Dempsey , A. Samani , F. Schrank , M. Shahryari , C. Warmuth , H. Tzschätzsch , J. Braun , I. Sack , Acta Biomater. 2021, 121, 393. 33326885 10.1016/j.actbio.2020.12.027

[89] K. J. Parker , Phys. Med. Biol. 2017, 62, 7425. 28766505 10.1088/1361-6560/aa8380

[90] L. E. Bilston , Comput. Methods Biomech. Biomed. Engin. 2002, 5, 283. 12186707 10.1080/10255840290032658

[91] G. Seano , H. T. Nia , K. E. Emblem , M. Datta , J. Ren , S. Krishnan , J. Kloepper , M. C. Pinho , W. W. Ho , M. Ghosh , V. Askoxylakis , G. B. Ferraro , L. Riedemann , E. R. Gerstner , T. T. Batchelor , P. Y. Wen , N. U. Lin , A. J. Grodzinsky , D. Fukumura , P. Huang , J. W. Baish , T. P. Padera , L. L. Munn , R. K. Jain , Nat. Biomed. Eng. 2019, 3, 230. 30948807 10.1038/s41551-018-0334-7 PMC6452896

[92] D. Greitz , R. Wirestam , A. Franck , B. Nordell , C. Thomsen , F. Stahlberg , Neuroradiol. 1992, 34, 370. 10.1007/BF00596493 1407513

[93] V. Kurtcuoglu , D. Poulikakos , Y. Ventikos , J. Biomech. Eng.‐Transactions of the ASME 2005, 127, 264. 10.1115/1.1865191 15971704

[94] A. Arani , H.‐K. Min , N. Fattahi , N. M. Wetjen , J. D. Trzasko , A. Manduca , C. R. Jack , K. H. Lee , R. L. Ehman , J. Huston , Magn. Reson. Med. 2018, 79, 1043. 28488326 10.1002/mrm.26738 PMC5811891

[95] S. Budday , R. Nay , R. de Rooij , P. Steinmann , T. Wyrobek , T. C. Ovaert , E. Kuhl , J. Mech. Behav. Biomed. Mater. 2015, 46, 318. 25819199 10.1016/j.jmbbm.2015.02.024 PMC4395547

[96] S. J. Lee , M. A. King , J. Sun , H. K. Xie , G. Subhash , M. Sarntinoranont , J. Mech. Behav. Biomed. Mater. 2014, 29, 213. 24099950 10.1016/j.jmbbm.2013.08.026 PMC8011428

[97] T. Kaster , I. Sack , A. Samani , J. Biomech. 2011, 44, 1158. 21329927 10.1016/j.jbiomech.2011.01.019

[98] S. Budday , G. Sommer , C. Birkl , C. Langkammer , J. Haybaeck , J. Kohnert , M. Bauer , F. Paulsen , P. Steinmann , E. Kuhl , G. A. Holzapfel , Acta Biomater. 2017, 48, 319. 27989920 10.1016/j.actbio.2016.10.036

[99] D. B. MacManus , B. Pierrat , J. G. Murphy , M. D. Gilchrist , J. Biomech. 2015, 48, 3213. 26189093 10.1016/j.jbiomech.2015.06.028

[100] S. Chatelin , A. Constantinesco , R. Willinger , Biorheology 2010, 47, 255. 21403381 10.3233/BIR-2010-0576

[101] D. Klatt , U. Hamhaber , P. Asbach , J. Braun , I. Sack , Phys. Med. Biol. 2007, 52, 7281. 18065839 10.1088/0031-9155/52/24/006

[102] F. Dittmann , S. Hirsch , H. Tzschatzsch , J. Guo , J. Braun , I. Sack , Magnetic Reson. Med. 2016, 76, 1116. 10.1002/mrm.26006 26485494

[103] J. Testu , M. D. J. McGarry , F. Dittmann , J. B. Weaver , K. D. Paulsen , I. Sack , E. E. W. Van Houten , J. Mech. Behav. Biomed. Mater. 2017, 74, 333. 28654854 10.1016/j.jmbbm.2017.06.027

[104] I. Sack , K. Joehrens , E. Wuerfel , J. Braun , Soft Matter 2013, 9, 5672.

[105] S. Hirsch , J. Braun , I. Sack , Magnetic Resonance Elastography, Physical Background And Medical Applications, Wiley‐VCH, Weinheim 2017.

[106] I. Sack , K. Jöhrens , J. Würfel , J. Braun , Soft Matter 2013, 9, 5672.

[107] K.‐J. Streitberger , L. Lilaj , F. Schrank , J. Braun , K.‐T. Hoffmann , M. Reiss‐Zimmermann , J. A. Käs , I. Sack , Proc. Natl. Acad. Sci. 2020, 117, 128. 31843897 10.1073/pnas.1913511116 PMC6955323

[108] M. Shahryari , H. Tzschätzsch , J. Guo , S. R. Marticorena Garcia , G. Böning , U. Fehrenbach , L. Stencel , P. Asbach , B. Hamm , J. A. Käs , J. Braun , T. Denecke , I. Sack , Cancer Res. 2019, 79, 5704. 31551364 10.1158/0008-5472.CAN-19-2150

[109] P. Asbach , S.‐R. Ro , N. Aldoj , J. Snellings , R. Reiter , J. Lenk , T. Köhlitz , M. Haas , J. Guo , B. Hamm , J. Braun , I. Sack , Invest Radiol. 2020, 55, 524. 32496317 10.1097/RLI.0000000000000685

[110] G. Bertalan , J. Becker , H. Tzschätzsch , A. Morr , H. Herthum , M. Shahryari , R. D. Greenhalgh , J. Guo , L. Schröder , C. Alzheimer , S. Budday , K. Franze , J. Braun , I. Sack , J. Mech. Behav. Biomed. Mater. 2023, 138, 105613. 36549250 10.1016/j.jmbbm.2022.105613

[111] J. Guo , G. Bertalan , D. Meierhofer , C. Klein , S. Schreyer , B. Steiner , S. Wang , R. Vieira da Silva , C. Infante‐Duarte , S. Koch , P. Boehm‐Sturm , J. Braun , I. Sack , Acta Biomater. 2019, 99, 433. 31449927 10.1016/j.actbio.2019.08.036

[112] N. Antonovaite , L. A. Hulshof , E. M. Hol , W. J. Wadman , D. Iannuzzi , J. Mech. Behav. Biomed. Mater. 2021, 113, 104159. 33137655 10.1016/j.jmbbm.2020.104159

[113] Y. Ryu , M. Iwashita , W. Lee , K. Uchimura , Y. Kosodo , Front. Aging Neurosci. 2021, 13, 709620. 34393762 10.3389/fnagi.2021.709620 PMC8361493

[114] G. McIlvain , H. Schwarb , N. J. Cohen , E. H. Telzer , C. L. Johnson , Dev. Cogn. Neurosci. 2018, 34, 27. 29906788 10.1016/j.dcn.2018.06.001 PMC6289278

[115] J. M. Schneider , G. McIlvain , C. L. Johnson , Dev. Neuropsychol. 2022, 47, 258. 35938379 10.1080/87565641.2022.2108425 PMC9397825

[116] H. Schwarb , C. L. Johnson , A. M. Daugherty , C. H. Hillman , A. F. Kramer , N. J. Cohen , A. K. Barbey , NeuroImage 2017, 153, 179. 28366763 10.1016/j.neuroimage.2017.03.061 PMC5637732

[117] T. Munder , A. Pfeffer , S. Schreyer , J. Guo , J. Braun , I. Sack , B. Steiner , C. Klein , J. Magn. Reson. Imaging 2018, 47, 105. 28422391 10.1002/jmri.25741

[118] G. Bertalan , J. Guo , H. Tzschätzsch , C. Klein , E. Barnhill , I. Sack , J. Braun , Magn. Reson. Med. 2019, 81, 2676. 30393887 10.1002/mrm.27586

[119] C. L. Johnson , M. D. J. McGarry , A. A. Gharibans , J. B. Weaver , K. D. Paulsen , H. Wang , W. C. Olivero , B. P. Sutton , J. G. Georgiadis , NeuroImage 2013, 79, 145. 23644001 10.1016/j.neuroimage.2013.04.089 PMC3676712

[120] A. Romano , J. Guo , T. Prokscha , T. Meyer , S. Hirsch , J. Braun , I. Sack , M. Scheel , Magn. Reson. Med. 2014, 72, 1755. 24347290 10.1002/mrm.25067

[121] M. McGarry , E. Van Houten , D. Sowinski , D. Jyoti , D. R. Smith , D. A. Caban‐Rivera , G. McIlvain , P. Bayly , C. L. Johnson , J. Weaver , K. Paulsen , Med. Image Anal. 2022, 78, 102432. 35358836 10.1016/j.media.2022.102432 PMC9122015

[122] J. L. Schmidt , D. J. Tweten , A. A. Badachhape , A. J. Reiter , R. J. Okamoto , J. R. Garbow , P. V. Bayly , J. Mech. Behav. Biomed. Mater . 2018, 79, 30. 29253729 10.1016/j.jmbbm.2017.11.045 PMC5807163

[123] E. Mace , I. Cohen , G. Montaldo , R. Miles , M. Fink , M. Tanter , IEEE Trans. Med. Imaging 2011, 30, 550. 20876009 10.1109/TMI.2010.2079940

[124] A. S. Morr , M. Nowicki , G. Bertalan , R. Vieira Silva , C. Infante Duarte , S. P. Koch , P. Boehm‐Sturm , U. Krügel , J. Braun , B. Steiner , J. A. Käs , T. Fuhs , I. Sack , Sci. Rep. 2022, 12, 16723. 36202964 10.1038/s41598-022-21105-7 PMC9537158

[125] E. G. Hain , C. Klein , T. Munder , J. Braun , K. Riek , S. Mueller , I. Sack , B. Steiner , PLoS One 2016, 11, e0161179. 27526042 10.1371/journal.pone.0161179 PMC4985068

[126] C. Klein , E. G. Hain , J. Braun , K. Riek , S. Mueller , B. Steiner , I. Sack , PLoS One 2014, 9, e92582. 24667730 10.1371/journal.pone.0092582 PMC3965445

[127] F. B. Freimann , S. Müller , K.‐J. Streitberger , J. Guo , S. Rot , A. Ghori , P. Vajkoczy , R. Reiter , I. Sack , J. Braun , NMR Biomed. 2013, 26, 1534. 23784982 10.1002/nbm.2987

[128] I. Sack , B. Beierbach , J. Wuerfel , D. Klatt , U. Hamhaber , S. Papazoglou , P. Martus , J. Braun , Neuroimage. 2009, 46, 652. 19281851 10.1016/j.neuroimage.2009.02.040

[129] P. L. Delgorio , L. V. Hiscox , A. M. Daugherty , F. Sanjana , R. T. Pohlig , J. M. Ellison , C. R. Martens , H. Schwarb , M. D. J. McGarry , C. L. Johnson , Cereb. Cortex 2021, 31, 2799. 33454745 10.1093/cercor/bhaa388 PMC8107787

[130] L. V. Hiscox , C. L. Johnson , M. D. J. McGarry , M. Perrins , A. Littlejohn , E. J. R. van Beek , N. Roberts , J. M. Starr , Neurobiol. Aging 2018, 65, 158. 29494862 10.1016/j.neurobiolaging.2018.01.010 PMC5883326

[131] A. Arani , M. C. Murphy , K. J. Glaser , A. Manduca , D. S. Lake , S. A. Kruse , C. R. Jack , R. L. Ehman , J. Huston , Neuroimage 2015, 111, 59. 25698157 10.1016/j.neuroimage.2015.02.016 PMC4387012

[132] M. C. Murphy , G. L. Curran , K. J. Glaser , P. J. Rossman , J. Huston , J. F. Poduslo , C. R. Jack , J. P. Felmlee , R. L. Ehman , Magn. Reson. Imaging 2011, 30, 535. 10.1016/j.mri.2011.12.019 PMC3433281 22326238

[133] M. C. Murphy , M. C. Huston 3rd, C. R. Jack Jr. , K. J. Glaser , A. Manduca , J. P. Felmlee , R. L. Ehman , J. Magn. Reson. Imaging 2011, 34, 494. 21751286 10.1002/jmri.22707 PMC3217096

[134] M. C. Murphy , D. T. Jones , C. R. Jack Jr., K. J. Glaser , M. L. Senjem , A. Manduca , J. P. Felmlee , R. E. Carter , R. L. Ehman , J. Huston III, Neuroimage Clin. 2015, 10, 283. 26900568 10.1016/j.nicl.2015.12.007 PMC4724025

[135] L. M. Gerischer , A. Fehlner , T. Köbe , K. Prehn , D. Antonenko , U. Grittner , J. Braun , I. Sack , A. Flöel , Neuroimage‐Clin. 2018, 18, 485. 29527504 10.1016/j.nicl.2017.12.023 PMC5842309

[136] T. Munder , A. Pfeffer , S. Schreyer , J. Guo , J. Braun , I. Sack , B. Steiner , C. Klein , J. Magn. Reson. Imaging 2018, 47, 105. 28422391 10.1002/jmri.25741

[137] K. Schregel , E. Wuerfel née Tysiak , P. Garteiser , I. Gemeinhardt , T. Prozorovski , O. Aktas , H. Merz , D. Petersen , J. Wuerfel , R. Sinkus , Proc. Natl. Acad. Sci. USA 2012, 109, 6650. 22492966 10.1073/pnas.1200151109 PMC3340071

[138] J. Weickenmeier , M. Kurt , E. Ozkaya , R. de Rooij , T. C. Ovaert , R. L. Ehman , K. Butts Pauly , E. Kuhl , J. Mech. Behav. Biomed. Mater. 2018, 84, 88. 29754046 10.1016/j.jmbbm.2018.04.009 PMC6751406

[139] G. Bertalan , C. Klein , S. Schreyer , B. Steiner , B. Kreft , H. Tzschätzsch , A. A. de Schellenberger , M. Nieminen‐Kelhä , J. Braun , J. Guo , I. Sack , Acta Biomater. 2020, 101, 395. 31726251 10.1016/j.actbio.2019.11.011

[140] Y.‐B. Lu , K. Franze , G. Seifert , C. Steinhäuser , F. Kirchhoff , H. Wolburg , J. Guck , P. Janmey , E.‐Q. Wei , J. Käs , A. Reichenbach , Proc. Natl. Acad. Sci. 2006, 103, 17759. 17093050 10.1073/pnas.0606150103 PMC1693820

[141] E. Moeendarbary , I. P. Weber , G. K. Sheridan , D. E. Koser , S. Soleman , B. Haenzi , E. J. Bradbury , J. Fawcett , K. Franze , Nat. Commun. 2017, 8, 14787. 28317912 10.1038/ncomms14787 PMC5364386

[142] E. P. Makhija , D. Espinosa‐Hoyos , A. Jagielska , K. J. Van Vliet , Neurosci. Lett. 2020, 717, 134673. 31838017 10.1016/j.neulet.2019.134673 PMC12023767

[143] J. Wuerfel , F. Paul , B. Beierbach , U. Hamhaber , D. Klatt , S. Papazoglou , F. Zipp , P. Martus , J. Braun , I. Sack , Neuroimage 2010, 49, 2520. 19539039 10.1016/j.neuroimage.2009.06.018

[144] K.‐J. Streitberger , I. Sack , D. Krefting , C. Pfüller , J. Braun , F. Paul , J. Wuerfel , PLoS One 2012, 7, e29888. 22276134 10.1371/journal.pone.0029888 PMC3262797

[145] A. Fehlner , J. R. Behrens , K.‐J. Streitberger , S. Papazoglou , J. Braun , J. Bellmann‐Strobl , K. Ruprecht , F. Paul , J. Würfel , I. Sack , J. Magn. Reson. Imaging 2016, 44, 51. 26714969 10.1002/jmri.25129

[146] J. M. Millward , J. Guo , D. Berndt , J. Braun , I. Sack , C. Infante‐Duarte , NMR Biomed. 2015, 28, 831. 25963743 10.1002/nbm.3319

[147] K. Riek , J. M. Millward , I. Hamann , S. Mueller , C. F. Pfueller , F. Paul , J. Braun , C. Infante‐Duarte , I. Sack , NeuroImage: Clin. 2012, 1, 81. 24179740 10.1016/j.nicl.2012.09.003 PMC3757734

[148] B. Geiger , A. Bershadsky , R. Pankov , K. M. Yamada , Nat. Rev. Mol. Cell Biol. 2001, 2, 793. 11715046 10.1038/35099066

[149] J. Madhusoodanan , Nature 2019, 566, 563. 30809069 10.1038/d41586-019-00681-1

[150] O. Chaudhuri , J. Cooper‐White , P. A. Janmey , D. J. Mooney , V. B. Shenoy , Nature 2020, 584, 535. 32848221 10.1038/s41586-020-2612-2 PMC7676152

[151] L. Bollmann , D. E. Koser , R. Shahapure , H. O. B. Gautier , G. A. Holzapfel , G. Scarcelli , M. C. Gather , E. Ulbricht , K. Franze , Front. Cell Neurosci. 2015, 9, 363. 26441534 10.3389/fncel.2015.00363 PMC4585148

[152] P. Moshayedi , G. Ng , J. C. F. Kwok , G. S. H. Yeo , C. E. Bryant , J. W. Fawcett , K. Franze , J. Guck , Biomaterials 2014, 35, 3919. 24529901 10.1016/j.biomaterials.2014.01.038

[153] P. Moshayedi , L. da F Costa , A. Christ , S. P. Lacour , J. Fawcett , J. Guck , K. Franze , J. Phys. Condens. Matter 2010, 22, 194114. 21386440 10.1088/0953-8984/22/19/194114

[154] A. S. G. van Oosten , X. Chen , L. Chin , K. Cruz , A. E. Patteson , K. Pogoda , V. B. Shenoy , P. A. Janmey , Nature 2019, 573, 96. 31462779 10.1038/s41586-019-1516-5

[155] K. Saha , A. J. Keung , E. F. Irwin , Y. Li , L. Little , D. V. Schaffer , K. E. Healy , Biophys. J. 2008, 95, 4426. 18658232 10.1529/biophysj.108.132217 PMC2567955

[156] N. Urban , I. M. Blomfield , F. Guillemot , Neuron. 2019, 104, 834. 31805262 10.1016/j.neuron.2019.09.026

[157] M. Segel , B. Neumann , M. F. E. Hill , I. P. Weber , C. Viscomi , C. Zhao , A. Young , C. C. Agley , A. J. Thompson , G. A. Gonzalez , A. Sharma , S. Holmqvist , D. H. Rowitch , K. Franze , R. J. M. Franklin , K. J. Chalut , Nature 2019, 573, 130. 31413369 10.1038/s41586-019-1484-9 PMC7025879

[158] J. Kjell , J. Fischer‐Sternjak , A. J. Thompson , C. Friess , M. J. Sticco , F. Salinas , J. Cox , D. C. Martinelli , J. Ninkovic , K. Franze , H. B. Schiller , M. Götz , Cell Stem Cell 2020, 26, 277. 32032526 10.1016/j.stem.2020.01.002 PMC7005820

[159] N. Urbán , I. M. Blomfield , F. Guillemot , Neuron 2019, 104, 834. 31805262 10.1016/j.neuron.2019.09.026

[160] T. Kobayashi , R. Kageyama , FEBS J. 2021, 288, 3082. 32902139 10.1111/febs.15555 PMC8246936

[161] Y. Ryu , M. Iwashita , W. Lee , K. Uchimura , Y. Kosodo , Front. Aging Neurosci. 2021, 13, 709620. 34393762 10.3389/fnagi.2021.709620 PMC8361493

[162] C. S. Batzdorf , A. S. Morr , G. Bertalan , I. Sack , R. V. Silva , C. Infante‐Duarte , Biol.‐Basel 2022, 11, 230. 10.3390/biology11020230 PMC8869215 35205095

[163] S. Wang , J. M. Millward , L. Hanke‐Vela , B. Malla , K. Pilch , A. Gil‐Infante , S. Waiczies , S. Mueller , P. Boehm‐Sturm , J. Guo , I. Sack , C. Infante‐Duarte , Front. Neurol. 2020, 10, 1382. 31998225 10.3389/fneur.2019.01382 PMC6970413

[164] R. V. Silva , K. Biskup , J. K. Zabala‐Jouvin , C. S. Batzdorf , C. Stellmach , A. S. Morr , I. Sack , A. Ludwig , V. Blanchard , C. Infante‐Duarte , Int. J. Biol. Macromol. 2023, 230, 123214. 36634800 10.1016/j.ijbiomac.2023.123214

[165] R. V. Silva , A. S. Morr , H. Herthum , S. P. Koch , S. Mueller , C. S. Batzdorf , G. Bertalan , T. Meyer , H. Tzschätzsch , A. A. Kühl , P. Boehm‐Sturm , J. Braun , M. Scheel , F. Paul , C. Infante‐Duarte , I. Sack , Acta Neuropathol. 2024, 147, 8. 38175305 10.1007/s00401-023-02658-x PMC10766667

[166] J. Braun , J. Bernarding , J. Snellings , T. Meyer , P. A. Dantas de Moraes , Y. Safraou , R. G. Wells , J. Guo , H. Tzschätzsch , A. Zappe , K. Pagel , I. M. Sauer , K. H. Hillebrandt , I. Sack , Acta Biomater. 2024, 10.1016/j.actbio.2024.05.007. 38729549

[167] E. Jonietz , Nature 2012, 491, S56. 23320288 10.1038/491s56a

[168] Y. A. Miroshnikova , J. K. Mouw , J. M. Barnes , M. W. Pickup , J. N. Lakins , Y. Kim , K. Lobo , A. I. Persson , G. F. Reis , T. R. McKnight , E. C. Holland , J. J. Phillips , V. M. Weaver , Nat. Cell Biol. 2016, 18, 1336. 27820599 10.1038/ncb3429 PMC5361403

[169] Y. Feng , E. H. Clayton , R. J. Okamoto , J. Engelbach , P. V. Bayly , J. R. Garbow , Phys. Med. Biol. 2016, 61, 6121. 27461395 10.1088/0031-9155/61/16/6121 PMC5943702

[170] K. M. Pepin , K. P. McGee , A. Arani , D. S. Lake , K. J. Glaser , A. Manduca , I. F. Parney , R. L. Ehman , J. Huston , AJNR Am. J. Neuroradiol. 2018, 39, 31. 29074637 10.3174/ajnr.A5415 PMC5766369

[171] K. Schregel , N. Nazari , M. O. Nowicki , M. Palotai , S. E. Lawler , R. Sinkus , P. E. Barbone , S. Patz , NMR Biomed. 2018, 31, e3840. 29193449 10.1002/nbm.3840 PMC6538416

[172] J. Guo , L. J. Savic , K. H. Hillebrandt , I. Sack , Invest Radiol. 2023, 58, 578. 36897804 10.1097/RLI.0000000000000971

[173] M. Reiss‐Zimmermann , K.‐J. Streitberger , I. Sack , J. Braun , F. Arlt , D. Fritzsch , K.‐T. Hoffmann , Clin. Neuroradiol. 2015, 25, 371. 24916129 10.1007/s00062-014-0311-9

[174] M. Simon , J. Guo , S. Papazoglou , H. Scholand‐Engler , C. Erdmann , U. Melchert , M. Bonsanto , J. Braun , D. Petersen , I. Sack , J. Wuerfel , The New J. Phys. 2013, 15, 085024.

[175] K.‐J. Streitberger , M. Reiss‐Zimmermann , F. B. Freimann , S. Bayerl , J. Guo , F. Arlt , J. Wuerfel , J. Braun , K.‐T. Hoffmann , I. Sack , PLoS One 2014, 9, e110588. 25338072 10.1371/journal.pone.0110588 PMC4206430

[176] A. Bertolotto , M. T. Giordana , M. L. Magrassi , A. Mauro , D. Schiffer , Acta Neuropathol. 1982, 58, 115. 6817587 10.1007/BF00691651

[177] A. Behin , K. Hoang‐Xuan , A. F. Carpentier , J. Y. Delattre , Lancet 2003, 361, 323. 12559880 10.1016/S0140-6736(03)12328-8

[178] P. G. Saffman , G. T. H. E. P. O. F. A. F. I. A. P. M. O. R. H.‐S. C. C. A. M. V. L. Taylor , Proceedings of the Royal Society of London Series a‐Mathematical and Physical Sciences 1958, 245, 312.

[179] K. Schregel , M. O. Nowicki , M. Palotai , N. Nazari , R. Zane , R. Sinkus , S. E. Lawler , S. Patz , Cancer Imaging. 2020, 20, 35. 32398076 10.1186/s40644-020-00314-1 PMC7218549

[180] L. Oswald , S. Grosser , D. M. Smith , J. A. Kas , J. Phys. D. Appl. Phys. 2017, 50, 483001. 29628530 10.1088/1361-6463/aa8e83 PMC5884432

[181] F. Sauer , A. Fritsch , S. Grosser , S. Pawlizak , T. Kießling , M. Reiss‐Zimmermann , M. Shahryari , W. C. Müller , K.‐T. Hoffmann , J. A. Käs , I. Sack , Soft Matter. 2021, 17, 10744. 34787626 10.1039/d1sm01291f PMC9386686

[182] B. Tully , Y. Ventikos , J. Fluid Mechanics 2011, 667, 188.

[183] E. Comellas , S. Budday , J. P. Pelteret , G. A. Holzapfel , P. Steinmann , Computer Methods in Applied Mechanics and Eng. 2020, 369, 113128.

[184] A. Ariza de Schellenberger , J. Bergs , I. Sack , M. Taupitz , In Quantification of Biophysical Parameters in Medical Imaging, (Eds.: Sack I. , Schaeffter T. ) Springer, Berlin 2017, chap. 6.

[185] J. J. Iliff , M. Wang , Y. Liao , B. A. Plogg , W. Peng , G. A. Gundersen , H. Benveniste , G. E. Vates , R. Deane , S. A. Goldman , E. A. Nagelhus , M. Nedergaard , Sci. Transl. Med. 2012, 4, 147ra111. 10.1126/scitranslmed.3003748 PMC3551275 22896675

[186] L. Lilaj , T. Fischer , J. Guo , J. Braun , I. Sack , S. Hirsch , Magn. Reson. Med. 2021, 85, 1655. 32902011 10.1002/mrm.28507

[187] G. R. Ge , J. P. Rolland , W. Song , M. Nedergaard , K. J. Parker , Phys. Med. Biol. 2023, 68, 95004. 10.1088/1361-6560/ad051f PMC10715715 37917188

[188] T. Boulet , M. L. Kelso , S. F. Othman , J. Neurotrauma 2013, 30, 1512. 23534701 10.1089/neu.2012.2788 PMC3751490

[189] A. M. Alfasi , A. V. Shulyakov , M. R. Del Bigio , J. Neurosurg. 2013, 119, 1255. 24032707 10.3171/2013.7.JNS121973

[190] K. Pogoda , L. Chin , P. C. Georges , F. J. Byfield , R. Bucki , R. Kim , M. Weaver , R. G. Wells , C. Marcinkiewicz , P. A. Janmey , New J. Phys. 2014, 16, 75002. 10.1088/1367-2630/16/7/075002 PMC4380293 25844043

[191] L. Juge , A. C. Pong , A. Bongers , R. Sinkus , L. E. Bilston , S. Cheng , PLoS One 2016, 11, e0148652. 26848844 10.1371/journal.pone.0148652 PMC4743852

[192] M. Wagshul , P. Eide , J. Madsen , Fluids and Barriers of the CNS 2011, 8, 5. 21349153 10.1186/2045-8118-8-5 PMC3042979

[193] T. Schubert , M. Pansini , O. Bieri , C. Stippich , S. Wetzel , S. Schaedelin , A. von Hessling , F. Santini , AJNR Am. J. Neuroradiol. 2015, 36, 562. 25395658 10.3174/ajnr.A4148 PMC8013071

[194] F. Schrank , C. Warmuth , H. Tzschätzsch , B. Kreft , S. Hirsch , J. Braun , T. Elgeti , I. Sack , J. Cereb. Blood Flow Metab. 2019, 40, 991. 31142226 10.1177/0271678X19850936 PMC7181097

[195] A. L. Adams , H. J. Kuijf , M. A. Viergever , P. R. Luijten , J. J. M. Zwanenburg , NMR Biomed. 2019, 32, e4050. 30575151 10.1002/nbm.4050 PMC6519010

[196] S. R. Mousavi , A. Fehlner , K. J. Streitberger , J. Braun , A. Samani , I. Sack , J. Biomech. 2014, 47, 1652. 24656483 10.1016/j.jbiomech.2014.02.038

[197] W. H. Bouvy , L. J. Geurts , H. J. Kuijf , P. R. Luijten , L. J. Kappelle , G. J. Biessels , J. J. Zwanenburg , NMR Biomed. 2016, 29, 1295. 25916399 10.1002/nbm.3306 PMC5008170

[198] S. Rashid , J. P. McAllister 2nd , Y. Yu , M. E. Wagshul , J. Cereb. Blood Flow Metab. 2012, 32, 318. 21934694 10.1038/jcbfm.2011.130 PMC3272598

[199] T. Meyer , B. Kreft , J. Bergs , E. Antes , M. S. Anders , B. Wellge , J. Braun , M. Doyley , H. Tzschätzsch , I. Sack , Front. Bioeng. Biotechnol. 2023, 11, 1140734. 37650041 10.3389/fbioe.2023.1140734 PMC10463728

[200] R. Forouhandehpour , M. Bernier , G. Gilbert , R. Butler , K. Whittingstall , E. Van Houten , Neuroimage: Reports 2021, 1, 100014. 10.1016/j.ynirp.2021.100014 PMC12172913 40567862

[201] A. Martín , E. Macé , R. Boisgard , G. Montaldo , B. Thézé , M. Tanter , B. Tavitian , J. Cereb. Blood Flow Metab. 2012, 32, 1496. 22491156 10.1038/jcbfm.2012.49 PMC3421095

[202] A. Hatt , S. Cheng , K. Tan , R. Sinkus , L. E. Bilston , AJNR Am. J. Neuroradiol. 2015, 36, 1971. 26045579 10.3174/ajnr.A4361 PMC7965045

[203] H. Herthum , M. Shahryari , H. Tzschatzsch , F. Schrank , C. Warmuth , S. Gorner , S. Hetzer , H. Neubauer , J. Pfeuffer , J. Braun , I. Sack , Front. Bioeng. Biotechnol. 2021, 9, 666456. 34026743 10.3389/fbioe.2021.666456 PMC8131519

[204] S. Hetzer , P. Birr , A. Fehlner , S. Hirsch , F. Dittmann , E. Barnhill , J. Braun , I. Sack , J. Cereb. Blood Flow Metab. 2018, 38, 116. 28151092 10.1177/0271678X17691530 PMC5757437

[205] S. Hetzer , F. Dittmann , K. Bormann , S. Hirsch , A. Lipp , D. J. Wang , J. Braun , I. Sack , J. Cereb. Blood Flow Metab. 2019, 39, 2445. 30182788 10.1177/0271678X18799241 PMC6893988

[206] S. Flogstad Svensson , E. Fuster‐Garcia , A. Latysheva , J. Fraser‐Green , W. Nordhoy , O. Isam Darwish , I. Thokle Hovden , S. Holm , E. O. Vik‐Mo , R. Sinkus , K. Eeg Emblem , Eur. J. Radiol. 2022, 147, 110136. 35007982 10.1016/j.ejrad.2021.110136

[207] A. Martín , E. Macé , R. Boisgard , G. Montaldo , B. Theze , M. Tanter , B. Tavitian , J. Cereb. Blood Flow Metab. 2012, 32, 1496. 22491156 10.1038/jcbfm.2012.49 PMC3421095

[208] K. Schregel , M. O. Nowicki , M. Palotai , N. Nazari , R. Zane , R. Sinkus , S. E. Lawler , S. Patz , Cancer Imaging 2020, 20, 35. 32398076 10.1186/s40644-020-00314-1 PMC7218549

[209] S. Patz , D. Fovargue , K. Schregel , N. Nazari , M. Palotai , P. E. Barbone , B. Fabry , A. Hammers , S. Holm , S. Kozerke , D. Nordsletten , R. Sinkus , Sci. Adv. 2019, 5, eaav3816. 31001585 10.1126/sciadv.aav3816 PMC6469937

[210] P. S. Lan , K. J. Glaser , R. L. Ehman , G. H. Glover , Neuroimage. 2020, 211, 116592. 32014553 10.1016/j.neuroimage.2020.116592 PMC7153752

[211] B. Kreft , H. Tzschätzsch , F. Schrank , J. Bergs , K.‐J. Streitberger , S. Wäldchen , S. Hetzer , J. Braun , I. Sack , Ultrasound Med. Biol. 2020, 46, 936. 32001088 10.1016/j.ultrasmedbio.2019.12.019

[212] G. Bertalan , P. Boehm‐Sturm , S. Schreyer , A.‐S. Morr , B. Steiner , H. Tzschätzsch , J. Braun , J. Guo , I. Sack , Acta Biomater. 2019, 96, 412. 31247381 10.1016/j.actbio.2019.06.034

[213] B. Kreft , H. Tzschätzsch , M. Shahryari , P. Haffner , J. Braun , I. Sack , K.‐J. Streitberger , Invest Radiol. 2022, 57, 77. 34380993 10.1097/RLI.0000000000000817

[214] M. Ascolese , A. Farina , A. Fasano , J. Biological Phys. 2019, 45, 379. 10.1007/s10867-019-09534-4 PMC6917688 31792778

[215] L. Kong , S. Qiu , Y. Chen , Z. He , P. Huang , Q. He , R.‐Y. Zhang , X.‐Q. Feng , L. Deng , Y. Li , F. Yan , G.‐Z. Yang , Y. Feng , NeuroImage 2023, 269, 119934. 36754123 10.1016/j.neuroimage.2023.119934

[216] S. Murase , N. Sakitani , T. Maekawa , D. Yoshino , K. Takano , A. Konno , H. Hirai , T. Saito , S. Tanaka , K. Shinohara , T. Kishi , Y. Yoshikawa , T. Sakai , M. Ayaori , H. Inanami , K. Tomiyasu , A. Takashima , T. Ogata , H. Tsuchimochi , S. Sato , S. Saito , K. Yoshino , Y. Matsuura , K. Funamoto , H. Ochi , M. Shinohara , M. Nagao , Y. Sawada , Nat. Biomed. Eng. 2023, 7, 1350. 37414976 10.1038/s41551-023-01061-x PMC10651490

[217] H. Herthum , S. Hetzer , M. Scheel , M. Shahryari , J. Braun , F. Paul , I. Sack , Acta Biomater. 2021, 138, 410. 34757062 10.1016/j.actbio.2021.10.038

[218] K. J. Streitberger , A. Fehlner , F. Pache , A. Lacheta , S. Papazoglou , J. Bellmann‐Strobl , K. Ruprecht , A. Brandt , J. Braun , I. Sack , F. Paul , J. Wuerfel , Eur. Radiol. 2017, 27, 2206. 27572811 10.1007/s00330-016-4561-6

[219] A. Lipp , C. Skowronek , A. Fehlner , K. J. Streitberger , J. Braun , I. Sack , Eur. Radiol. 2018, 28, 3347. 29460073 10.1007/s00330-017-5269-y

[220] A. Lipp , R. Trbojevic , F. Paul , A. Fehlner , S. Hirsch , M. Scheel , C. Noack , J. Braun , I. Sack , Neuroimage Clin. 2013, 3, 381. 24273721 10.1016/j.nicl.2013.09.006 PMC3814959

[221] G. R. Huesmann , H. Schwarb , D. R. Smith , R. T. Pohlig , A. T. Anderson , M. D. J. McGarry , K. D. Paulsen , T. M. Wszalek , B. P. Sutton , C. L. Johnson , Neuroimage Clin. 2020, 27, 102313. 32585569 10.1016/j.nicl.2020.102313 PMC7322100

[222] A. Fehlner , J. R. Behrens , K.‐J. Streitberger , S. Papazoglou , J. Braun , J. Bellmann‐Strobl , K. Ruprecht , F. Paul , J. Würfel , I. Sack , J. Magn. Reson. Imaging 2016, 44, 51. 26714969 10.1002/jmri.25129

[223] K.‐J. Streitberger , A. Fehlner , F. Pache , et al., Eur. Radiology. 2017, 27, 2206. 10.1007/s00330-016-4561-6 27572811

[224] K.‐J. Streitberger , E. Wiener , J. Hoffmann , F. B. Freimann , D. Klatt , J. Braun , K. Lin , J. McLaughlin , C. Sprung , R. Klingebiel , I. Sack , NMR Biomed. 2011, 24, 385. 20931563 10.1002/nbm.1602

[225] A. Lipp , C. Skowronek , A. Fehlner , K.‐J. Streitberger , J. Braun , I. Sack , Eur. Radiol. 2018, 28, 3347. 29460073 10.1007/s00330-017-5269-y

[226] A. Lipp , R. Trbojevic , F. Paul , A. Fehlner , S. Hirsch , M. Scheel , C. Noack , J. Braun , I. Sack , Neuroimage Clin. 2013, 3, 381. 24273721 10.1016/j.nicl.2013.09.006 PMC3814959

[227] P. Karki , M. C. Murphy , P. M. Cogswell , M. L. Senjem , J. Graff‐Radford , B. D. Elder , A. Perry , C. S. Graffeo , F. B. Meyer , C. R. Jack , R. L. Ehman , J. Huston , AJNR Am. J. Neuroradiol. 2024, 45, 328. 38272572 10.3174/ajnr.A8108 PMC11286123

[228] M. ElSheikh , A. Arani , A. Perry , B. F. Boeve , F. B. Meyer , R. Savica , R. L. Ehman , J. Huston , AJR Am. J. Roentgenol. 2017, 209, 403. 28570101 10.2214/AJR.16.17455 PMC5597304

[229] M. C. Murphy , P. M. Cogswell , J. D. Trzasko , A. Manduca , M. L. Senjem , F. B. Meyer , R. L. Ehman , J. 3rd Huston , Invest Radiol. 2020, 55, 200. 32058331 10.1097/RLI.0000000000000630 PMC7681913

[230] A. Perry , C. S. Graffeo , N. Fattahi , M. M. ElSheikh , N. Cray , A. Arani , R. L. Ehman , K. J. Glaser , A. Manduca , F. B. Meyer , J. 3rd Huston , World Neurosurg. 2017, 99, 695. 28063896 10.1016/j.wneu.2016.12.121 PMC5357459

[231] F. B. Freimann , K.‐J. Streitberger , D. Klatt , K. Lin , J. McLaughlin , J. Braun , C. Sprung , I. Sack , Neuroradiology 2012, 54, 189. 21538046 10.1007/s00234-011-0871-1

[232] K.‐J. Streitberger , E. Wiener , J. Hoffmann , F. B. Freimann , D. Klatt , J. Braun , K. Lin , J. McLaughlin , C. Sprung , R. Klingebiel , I. Sack , NMR Biomed. 2011, 24, 385. 20931563 10.1002/nbm.1602

[233] L. M. Solamen , M. D. J. McGarry , J. Fried , J. B. Weaver , S. S. Lollis , K. D. Paulsen , Acad. Radiol. 2021, 28, 457. 32331966 10.1016/j.acra.2020.03.009 PMC7575616

[234] A. Kolipaka , P. A. Wassenaar , S. Cha , W. M. Marashdeh , X. Mo , P. Kalra , B. Gans , B. Raterman , E. Bourekas , Clin. Imaging. 2018, 51, 114. 29459315 10.1016/j.clinimag.2018.02.005 PMC6087505

[235] F. B. Freimann , K.‐J. Streitberger , D. Klatt , K. Lin , J. McLaughlin , J. Braun , C. Sprung , I. Sack , Neuroradiol. 2012, 54, 189. 10.1007/s00234-011-0871-1 21538046

[236] I. Sack , K. J. Streitberger , D. Krefting , F. Paul , J. Braun , PlosOne 2011, 6, e23451. 10.1371/journal.pone.0023451 PMC3171401 21931599

[237] C. L. Johnson , H. Schwarb , K. M. Horecka , M. D. J. McGarry , C. H. Hillman , A. F. Kramer , N. J. Cohen , A. K. Barbey , NeuroImage 2018, 171, 99. 29317306 10.1016/j.neuroimage.2018.01.007 PMC5857428

[238] S. Hetzer , S. Hirsch , J. Braun , I. Sack , M. Weygandt , Brain Imaging Behav. 2019, 14, 2477. 10.1007/s11682-019-00200-w 31512097

[239] A. Bunevicius , K. Schregel , R. Sinkus , A. Golby , S. Patz , Neuroimage Clin 2020, 25, 102109. 31809993 10.1016/j.nicl.2019.102109 PMC6909210

[240] J. D. Hughes , N. Fattahi , J. Van Gompel , A. Arani , F. Meyer , G. Lanzino , M. J. Link , R. Ehman , J. Huston , Neurosurgery 2015, 77, 653. 26197204 10.1227/NEU.0000000000000892 PMC4749919

[241] I. Sack , B. Beierbach , J. Wuerfel , D. Klatt , U. Hamhaber , S. Papazoglou , P. Martus , J. Braun , NeuroImage 2009, 46, 652. 19281851 10.1016/j.neuroimage.2009.02.040

[242] L. M. Gerischer , A. Fehlner , T. Köbe , K. Prehn , D. Antonenko , U. Grittner , J. Braun , I. Sack , A. Flöel , NeuroImage: Clin. 2018, 18, 485. 29527504 10.1016/j.nicl.2017.12.023 PMC5842309

