# Supplementary material for: The Networking Brain: How Extracellular Matrix, Cellular Networks, and Vasculature Shape the In Vivo Mechanical Properties of the Brain
Source: Adv Sci (Weinh). 2024 Jun 14;11(31):2402338. doi: 10.1002/advs.202402338 (PMC11336943; doi:10.1002/advs.202402338)
Supplement: Supplementary file 1 — Supporting Information [file ADVS-11-2402338-s001.docx]

Supporting Information

The networking brain: how extracellular matrix, cellular networks, and vasculature shape the in vivo mechanical properties of the brain

Judith Bergs†, Anna S. Morr†, Rafaela V. Silva, Carmen Infante-Duarte, Ingolf Sack*

† These authors have contributed equally to this work and share first authorship.

**Table S1.** Brain networks, their macro-organization, involved structures and connections with other networks.

| **Network** | **Macro-organization** | **Structures and their mechanical functions** | **Inter-network connections** |
| --- | --- | --- | --- |
| Neurons | **Total brain**  Weight: 1509 ± 299 g^[1]^  67-86 billion neurons ^[1-3]^  **Cerebral cortex**  Weight: 1233 ± 234 g^[1]^  81.8% of brain mass, contains 19.0% of brain neurons^[1]^  10-20 billion neurons^[1],[2]^  **Grey matter**  Weight: 316 ± 53 g^[1]^  15 ± 2 billion neurons^[1]^  Neuron density per mm^2^: Layer III: 419, layer V: 454, layer VI: 458^[4]^  **White matter**  Weight: 294 ± 60 g^[1]^  1.29 ± 0.54 billion neurons^[1]^  Neuron density (not uniformly distributed): 250-1000 per mm^3 [5]^  Fiber (myelinated axons) diameters ranging from 0.16-9µm^[6]^  **Cerebellum**  154 ± 19 g^[1]^  10.3% of brain mass, 80.2% of brain neurons^[1]^  54-105 billion neurons^[1,2]^  Density: 721 ± 69 cells (neurons + glial cells + pericytes) per 0,001 mm³ in cerebellar cortex^[7]^ | - Myelin: extracellular support - Cytoskeleton: intracellular scaffold and support, cell movement - Cytoplasm: intracellular pressure, cell viscosity, (in)compressibility - Organelles: scaffold | - Other neurons – through synapses - Glial cells – through myelin (oligodendrocytes), perisynaptic astrocytic processes (astrocytes) - ECM through via filopodia and glycocalyx - Vasculature |
| Glial cells | **Total brain**  Estimated 40-50 billion glial cells^[2]^:   - Oligodendrocytes 29-75%^[2]^ - Astrocytes 19-40%^[2]^ - Microglia ≤ 10%^[2]^   **Cerebral cortex**  18.2-100 billion non-neuronal cells (including glial and endothelial cells), of which around 70% are estimated to be glial cells^[2]^  **Grey matter**  18.2-39 billion^[2]^  Density: 100,000 per mm^3^ ^[2]^  **White matter**  20,000-200,000 per mm^3^, mostly oligodendrocytes^[2]^  **Cerebellum**  3 billion^[2]^ | - Cytoskeleton: intracellular scaffold and support, cell movement - Cytoplasm: intracellular pressure, cell viscosity, (in)compressibility - Organelles: scaffold | - Neurons – through myelin (oligodendrocytes), perisynaptic astrocytic processes (astrocytes) - Other glial cells - ECM – through glycocalyx - Vasculature - through endfeet via ECM and cell-adhesion molecules |
| Extracellular space (ECS) | **Extracellular/ interstitial space (ECS/ ISS) = interstitial fluid + extracellular matrix (ISF + ECM)**  15-20% of brain volume^[8]^, about 250-300 ml^[9]^  During sleep increased to 22-24%^[10]^  ECM includes:   - Interstitial ECM - Perineuronal ECM (perineuronal nets, PNNs) - Perivascular ECM (basal lamina)   **Cerebrospinal fluid (CSF) + interstitial fluid (ISF)**  8.7% for grey matter (GM)^[11]^  5.9% for white matter (WM)^[11]^  **CSF**  150 mL, 25mL in ventricles and 100 mL in subarachnoid spaces^[12]^  <2% (0.57 ml) for WM^[13]^  7.5% (10.8 ml) for GM with some older subjects 25% in GM^[13]^  3.7% (15.3 ml) for mixed brain tissue^[13]^ | - Scaffold for RGC migration (CNS development) - Reservoir for ions and nutrients - Hydrophilic interactions: water binding, elasticity - Hydrophobic interactions: mechanical attenuation, viscosity, fluidity - ISF and CSF are interchanging via glymphatic system: clearance of metabolites (active flow) - Marginal intracranial pressure (ICP) regulation (fluid excess expelled via CSF) | - Neurons – glycocalyx, cell adhesion molecules (e.g. integrins) - Glial cells – glycocalyx, cell adhesion molecules e.g. integrins) - Other ECM components - Vasculature – glycocalyx, cell adhesion molecules (e.g. integrins), tight junctions, primary cilia - Basal lamina – radial glial cells (RGCs) through endfeet |
| Vasculature | **Vascular system:** 3-5% of brain volume^[8]^  **Arterial cererebral blood volume (CBVa)**  20-30% of intravascular space occupied by all blood vessels (CBVtot), can increase by up to 80% of its baseline values during functional or physiological stimulation^[14]^  *whole brain*  0.70 - 1.00 mL/ 100 mL (MRI)^[14,15]^ and 2.60 mL/ 100 mL (PET)^[14,16]^  *GM*  0.67-1.4 mL/ 100 mL (MRI) and 3.21 mL/ 100 mL (PET)  *WM*  0.46 - 0.64 mL / 100 mL (MRI) or 1.87 mL/ 100 mL (PET)^[14]^  **Venous blood volume**  *Whole brain*  2.46 - 3.77 mL/ 100 mL (MRI)  *GM*  1.75 – 5.5 mL / 100 mL (MRI)  *WM*  0.58 – 3.9 mL / 100 mL (MRI)^[14]^  **Diameters**  *arteries*  Central arteries: 260 – 280 µm^[17]^  Peripheral arteries: 150 – 180 µm^[17]^  Penetrating arteries: 40 µm^[17]^  *Arterioles*  Anastomoses: 25 – 90 µm^[17]^  Small straight arterioles: 10 µm^[17]^  *Veins*  Central veins: 280-380 µm^[17]^  Peripheral veins: 130 µm^[17]^  Anastomoses: up to 180 µm^[17]^  *Capillaries*  3-7 µm^[18]^  **Endothelial cells**  *Whole brain*  25%-30% of non-neuronal cells (^[2]^)  *white matter*  10-20% of non-neuronal cells^[2]^  *Cerebellum*  ~13 billion endothelial cells^[2]^  **Densities**  Vascular density (pre-frontal + basal forebrain + motor/sensory + hippocampus): 89.8 per mm^2^ ^[19]^  Cerebral arteries: 1.378 g/cm^3^ ^[20]^  Cerebral venous sinuses: 1.130 g/ cm^3^ ^[20]^ | - Providing of brain parenchyma with nutrients, physiological regulation: vascular walls, vessel diameter, vessel density, branching, tortuosity, arterial pulsation, blood pressure, osmotic pressure, vascular constriction / dilatation | - Astrocytes - through endfeet via ECM and cell-adhesion molecules - ECM – through vascular basement membrane |

**References**

[1]. Azevedo FA, Carvalho LR, Grinberg LT, Farfel JM, Ferretti RE, Leite RE, Jacob Filho W, Lent R, Herculano-Houzel S. Equal numbers of neuronal and nonneuronal cells make the human brain an isometrically scaled-up primate brain. *J Comp Neurol*. Apr 10 2009;513(5):532-41. doi:10.1002/cne.21974

[2]. von Bartheld CS, Bahney J, Herculano-Houzel S. The search for true numbers of neurons and glial cells in the human brain: A review of 150 years of cell counting. *J Comp Neurol*. Dec 15 2016;524(18):3865-3895. doi:10.1002/cne.24040

[3]. Andrade-Moraes CH, Oliveira-Pinto AV, Castro-Fonseca E, da Silva CG, Guimaraes DM, Szczupak D, Parente-Bruno DR, Carvalho LR, Polichiso L, Gomes BV, Oliveira LM, Rodriguez RD, Leite RE, Ferretti-Rebustini RE, Jacob-Filho W, Pasqualucci CA, Grinberg LT, Lent R. Cell number changes in Alzheimer's disease relate to dementia, not to plaques and tangles. *Brain*. Dec 2013;136(Pt 12):3738-52. doi:10.1093/brain/awt273

[4]. Trapp BD, Vignos M, Dudman J, Chang A, Fisher E, Staugaitis SM, Battapady H, Mork S, Ontaneda D, Jones SE, Fox RJ, Chen J, Nakamura K, Rudick RA. Cortical neuronal densities and cerebral white matter demyelination in multiple sclerosis: a retrospective study. *Lancet Neurol*. Oct 2018;17(10):870-884. doi:10.1016/S1474-4422(18)30245-X

[5]. von Bartheld CS, Bahney J, Herculano-Houzel S. The search for true numbers of neurons and glial cells in the human brain: A review of 150 years of cell counting. *Journal of Comparative Neurology*. 2016;524(18):3865-3895. doi:<https://doi.org/10.1002/cne.24040>

[6]. Liewald D, Miller R, Logothetis N, Wagner HJ, Schuz A. Distribution of axon diameters in cortical white matter: an electron-microscopic study on three human brains and a macaque. *Biol Cybern*. Oct 2014;108(5):541-57. doi:10.1007/s00422-014-0626-2

[7]. Lange W. Cell number and cell density in the cerebellar cortex of man and some other mammals. *Cell Tissue Res*. 1975;157(1):115-24.

[8]. Lei Y, Han H, Yuan F, Javeed A, Zhao Y. The brain interstitial system: Anatomy, modeling, in vivo measurement, and applications. *Progress in neurobiology*. 2017;157:230-246.

[9]. Segal MB. Extracellular and cerebrospinal fluids. *Journal of Inherited Metabolic Disease*. 1993;16(4):617-638. doi:<https://doi.org/10.1007/BF00711896>

[10]. DiNuzzo M, Nedergaard M. Brain energetics during the sleep-wake cycle. *Curr Opin Neurobiol*. Dec 2017;47:65-72. doi:10.1016/j.conb.2017.09.010

[11]. Bender B, Klose U. Cerebrospinal fluid and interstitial fluid volume measurements in the human brain at 3T with EPI. *Magn Reson Med*. Apr 2009;61(4):834-41. doi:10.1002/mrm.21915

[12]. Sakka L, Coll G, Chazal J. Anatomy and physiology of cerebrospinal fluid. *European Annals of Otorhinolaryngology, Head and Neck Diseases*. 2011/12/01/ 2011;128(6):309-316. doi:<https://doi.org/10.1016/j.anorl.2011.03.002>

[13]. Ernst T, Kreis R, Ross BD. Absolute Quantitation of Water and Metabolites in the Human Brain. I. Compartments and Water. *Journal of Magnetic Resonance, Series B*. 1993/08/01/ 1993;102(1):1-8. doi:<https://doi.org/10.1006/jmrb.1993.1055>

[14]. Hua J, Liu P, Kim T, Donahue M, Rane S, Chen JJ, Qin Q, Kim SG. MRI techniques to measure arterial and venous cerebral blood volume. *Neuroimage*. Feb 16 2018;doi:10.1016/j.neuroimage.2018.02.027

[15]. An H, Lin W. Cerebral oxygen extraction fraction and cerebral venous blood volume measurements using MRI: effects of magnetic field variation. *Magn Reson Med*. May 2002;47(5):958-66. doi:10.1002/mrm.10148

[16]. Heijtel DF, Petersen ET, Mutsaerts HJ, Bakker E, Schober P, Stevens MF, van Berckel BN, Majoie CB, Booij J, van Osch MJ, van Bavel ET, Boellaard R, Lammertsma AA, Nederveen AJ. Quantitative agreement between [(15)O]H2O PET and model free QUASAR MRI-derived cerebral blood flow and arterial blood volume. *NMR Biomed*. Apr 2016;29(4):519-26. doi:10.1002/nbm.3480

[17]. Duvernoy HM, Delon S, Vannson JL. Cortical blood vessels of the human brain. *Brain Res Bull*. Nov 1981;7(5):519-79.

[18]. Betz AL, Goldstein GW. Brain Capillaries. In: Lajtha A, ed. *Structural Elements of the Nervous System*. Springer US; 1984:465-484.

[19]. Fischer VW, Siddiqi A, Yusufaly Y. Altered angioarchitecture in selected areas of brains with Alzheimer's disease. *Acta Neuropathol*. 1990;79(6):672-9.

[20]. Barber TW, Brockway JA, Higgins LS. The density of tissues in and about the head. *Acta Neurol Scand*. 1970;46(1):85-92.
